# The glycosylation deficiency of flavivirus NS1 attenuates virus replication through interfering with the formation of viral replication compartments

**DOI:** 10.1186/s12929-024-01048-z

**Published:** 2024-06-07

**Authors:** Shuhan Huang, Pan-Deng Shi, Xiao-Xuan Fan, Yang Yang, Cheng-Feng Qin, Hui Zhao, Lei Shi, Yali Ci

**Affiliations:** 1 State Key Laboratory of Common Mechanism Research for Major Diseases, Institute of Basic Medical Sciences, Chinese Academy of Medical Sciences; and School of Basic Medicine, Peking Union Medical College, Beijing, 100005 China; 2 Department of Biochemistry and Molecular Biology, Institute of Basic Medical Sciences, Chinese Academy of Medical Sciences; and School of Basic Medicine, Peking Union Medical College, Beijing, 100005 China; 3 State Key Laboratory of Pathogen and Biosecurity, Beijing Institute of Microbiology and Epidemiology, Academy of Military Medical Sciences, Beijing, China

**Keywords:** Flavivirus, NS1, Glycosylation, Replication compartments, ER remodeling

## Abstract

**Background:**

Flavivirus is a challenge all over the world. The replication of flavivirus takes place within membranous replication compartments (RCs) derived from endoplasmic reticulum (ER). Flavivirus NS1 proteins have been proven essential for the formation of viral RCs by remodeling the ER. The glycosylation of flavivirus NS1 proteins is important for viral replication, yet the underlying mechanism remains unclear.

**Methods:**

HeLa cells were used to visualize the ER remodeling effects induced by NS1 expression. ZIKV replicon luciferase assay was performed with BHK-21 cells. rZIKV was generated from BHK-21 cells and the plaque assay was done with Vero Cells. Liposome co-floating assay was performed with purified NS1 proteins from 293T cells.

**Results:**

We found that the glycosylation of flavivirus NS1 contributes to its ER remodeling activity. Glycosylation deficiency of NS1, either through N-glycosylation sites mutations or tunicamycin treatment, compromises its ER remodeling activity and interferes with viral RCs formation. Disruption of NS1 glycosylation results in abnormal aggregation of NS1, rather than reducing its membrane-binding activity. Consequently, deficiency in NS1 glycosylation impairs virus replication.

**Conclusions:**

In summary, our results highlight the significance of NS1 glycosylation in flavivirus replication and elucidate the underlying mechanism. This provides a new strategy for combating flavivirus infections.

**Supplementary Information:**

The online version contains supplementary material available at 10.1186/s12929-024-01048-z.

## Introduction

Flaviviruses are a family of positive-sense, single-stranded RNA viruses, comprising more than 70 members, including Zika virus (ZIKV), dengue virus (DENV), yellow fever virus (YFV), and Japanese Encephalitis virus (JEV), among others [1]. Many flaviviruses, such as ZIKV and DENV, are primarily transmitted by *Aedes aegypti* and *A. albopictus* mosquitoes, posing significant public health concerns due to their potential for widespread infection and serious clinical manifestations in severe cases [2, 3].

While most individuals infected with ZIKV experience only subclinical or mild influenza-like symptoms, ZIKV infection can lead to neurological syndromes, including Guillain–Barré syndrome, characterized by an autoimmune attack on peripheral nerves. Additionally, ZIKV infection during pregnancy may transmit to the fetus, causing microcephaly [4, 5]. DENV is a public health concern due to its prevalence in more than 100 countries, with an estimated 390 million people infected worldwide and 96 million manifested cases annually [6, 7]. Common symptoms of DENV infection include high fever, headache, body aches, nausea and rash. In some cases, dengue fever can be severe and fatal [8]. Despite increased research efforts, specific antiviral treatments and safe, effective vaccines for flaviviruses are still lacking [9–11].

Flavivirus virions are small, spherical particles with a diameter of 40 ~ 70 nm. The RNA genome, approximately 11kb in size, encodes a precursor polyprotein that is cleaved into three structural proteins (capsid, precursor membrane and envelope) and seven non-structural (NS) proteins (NS1, NS2A, NS2B, NS3, NS4A, NS4B and NS5) by viral NS2B3 protease and host proteases. Three structural proteins, along with genomic RNA, assemble within a host envelope to form new virus particles. Seven NS proteins play roles in viral RNA replication, virion assembly, and modulation of host immune responses [12–14].

Flavivirus replication occurs within specialized membranous structures known as replication compartments (RCs), derived from the endoplasmic reticulum (ER) [15, 16]. Recent research has illuminated the molecular mechanisms governing the formation of the flavivirus RCs. It has been shown that virus-induced rearrangement of ER membranes is critical for the formation of RCs [16]. NS1, NS4A, and NS4B have all been reported to possess the ability to remodel the ER. NS4A facilitates membrane association and contributes to membrane remodeling [17, 18], while NS4B interacts with NS1 or host protein TMEM41B to induce membrane curvature [19, 20]. In our previous study, we found that ZIKV NS1 is indispensable for the establishment of RCs, depending on its membrane remodeling capacity [15, 21].

Flavivirus NS1 is a conserved, glycosylated protein residing in the ER lumen, with a molecular weight ranging from 46 ~ 55 kDa based on its glycosylation level [22]. Flavivirus NS1 exists in various oligomeric forms. Intracellularly, NS1 primarily exists as a dimer, while secretory NS1 forms tetramers or hexamers, comprising two or three NS1 dimers, respectively [23, 24]. The dimeric NS1 consists of a central β-ladder domain, a hydrophobic β-roll domain, and two wing domains [25]. Most flaviviruses have two conserved N-glycosylation sites in NS1 proteins, N130 and N207 [26]. N130 is located in the wing domain, while N207 is located in the continuous β-ladder domain [25, 27]. NS1 undergoes glycosylation at these sites with high mannose glycans, with the glycans at N130 further modified into complex glycans in the Golgi apparatus by Golgi-resident enzymes [28]. Evidence from previous studies indicates the importance of NS1 glycosylation for flavivirus replication. For example, Tajima et al. and Pryor et al. declared that N130 glycosylation of DENV is essential for the production of progeny virus [29, 30]. Crabtree et al. showed that deglycosylation of DENV2 (16681) at N207 site can affect the growth and pathogenicity of the virus [31]. Additionally, double mutations in these glycosylation sites on ZIKV NS1 result in reduced replication and smaller cell plaques [32]. In summary, substantial evidence supports that NS1 glycosylation is important for flavivirus replication. However, how does glycosylation affect NS1 function and subsequently impair viral replication remains unclear.

Based on our previous study demonstrating the critical role of ZIKV NS1 in viral RCs formation through ER membrane rearrangement [21], we sought to investigate whether the glycosylation of flavivirus NS1 is involved in ER remodeling and viral RCs formation. In this study, we found that the deficiency of NS1 glycosylation, whether through point mutations or tunicamycin treatment, indeed impairs ER remodeling, subsequently interfering with viral RCs formation. Mechanistically, disruption of glycosylation leads to the abnormal aggregation of NS1, rather than reducing its membrane binding activity, resulting in its partially inactivation. Thus, glycosylation deficiency of NS1 impairs ZIKV replication and the production of infectious virions.

## Materials and methods

### Cells & viruses

HeLa (ATCC CCL-2), HEK293T (ATCC CRL3216), and Vero (ATCC CCL-81) cells were cultured in DMEM (Gibco), supplemented with 10% fetal bovine serum (FBS, biosera). BHK-21 cells (ATCC CCL10) were cultured in DMEM (Gibco), supplemented with 5% FBS. All cells were cultured with 5% CO_2_ at 37°C. Zika virus strain (FSS13025) and DENV-2 virus strain (DENV2_China_SZ_2015) were gifts from State Key Laboratory of Pathogen and Biosecurity, Beijing, China.

### Antibodies & reagents

Antibodies used for Western blotting include Myc (MBL, Cat. #M192-3), ZIKV/DENV NS1 (BioFront, Cat. # B-1191–46), Calnexin (MBL, Cat. #M178-3). Antibodies used for immunofluorescence include Myc (MBL, Cat. #M192-3), climp63 (Proteintech, Cat. #16686-1-AP), ZIKV/DENV NS1 (BioFront, Cat. #B-1191–46), dsRNA-J2 (Scicons), Goat anti-mouse IgG with Alexa Fluor 488 (Invitrogen). Reagents include tunicamycin (MCE, Cat. #HY-A0098), Ribonuclease A (Takara, Cat. #2158).

### Clone construction

pcDNA3.1-ss-Myc ZIKV/DENV NS1 has been described elsewhere [21]. It has an N-terminal bovine preprolactin signal peptide sequence followed by a Myc tag upstream of the NS1 coding sequence. ZIKV/DENV NS1 mutants were produced by point mutations at N130, N207 sites on pcDNA3.1-ss-Myc ZIKV/DENV NS1 vector referring to QuikChange® II Site-Directed Mutagenesis manual. pcDNA3.1-ss-Myc ZIKV NS1-Twin-Strep clones (WT and mutants) were generated by adding a Twin-Strep-Tag at the C-terminus of NS1 for proteins purification. All plasmids were validated by DNA sequencing.

### Immunofluorescence

HeLa or BHK cells were seeded on the coverslips in 24-well plates. Cells were fixed with 4% paraformaldehyde for 10 min at RT. Cells were then washed with PBS three times and permeabilized with 0.2% Triton-X100 for 10 min at RT. After being blocked with 3% Bovine Serum Albumin (BSA), cells were incubated with the primary antibodies, Myc (MBL, 1:500) and climp63 (Proteintech, 1:300) or ZIKV/DENV NS1 (BioFront, 1:200) for 2 h at 37 °C. After three PBS washes, cells were incubated with the secondary antibodies for 1 h at 37 °C. The cells were stained with DAPI (1 μg/mL DAPI in PBS) for 10 min and then washed with PBS. Coverslips with cells were mounted using ProLong™ Diamond Antifade Mountant (Invitrogen Cat. #P36970). Fluorescent images were captured by Leica TCS SP8 gSTED 3X microscope with confocal mode. Particularly, extracellular RNA was digested with Ribonuclease A at 37 °C for 30 min before cell fixation for dsRNA staining.

### Protein expression and purification

HEK293T cells were transfected with pcDNA3.1-ss-Myc ZIKV NS1-Twin-Strep (WT or mutants) plasmids by PEI. After 24 h, Cells were lysed by RIPA buffer with protease inhibitors cocktail (1 mM PMSF, 1 μg/mL Aprotinin, 1 μg/mL Pepstatin, and 1 μg/mL Leupeptin), and the cell lysate was passed through a low temperature ultra high pressure cell crusher (JNBIO, China) twice. The cell lysate was then centrifuged for 15 min at 20,000 rpm, 4 °C. The supernatant was incubated with Streptactin Beads 4FF (Smart-Lifesciences) for 4 h at 4 °C. Beads were collected and washed with washing buffer (50 mM Tris pH = 7.4, 100 mM NaCl, 1 mM DTT). The proteins were eluted by elution buffer (50 mM Tris pH = 7.4, 100 mM NaCl, 10% glycerol, 1 mM DTT, 5 mM biotin).

### TEM

Cells were seeded in 35-mm dishes. After transfection with indicated plasmids or virus infections, cells were fixed with 2.5% glutaraldehyde in PBS. Sample preparation was performed at the Center of Biomedical Analysis, Tsinghua University. TEM images were obtained using electron microscope H-7650.

### Protein quantification

**Fig. 1:**
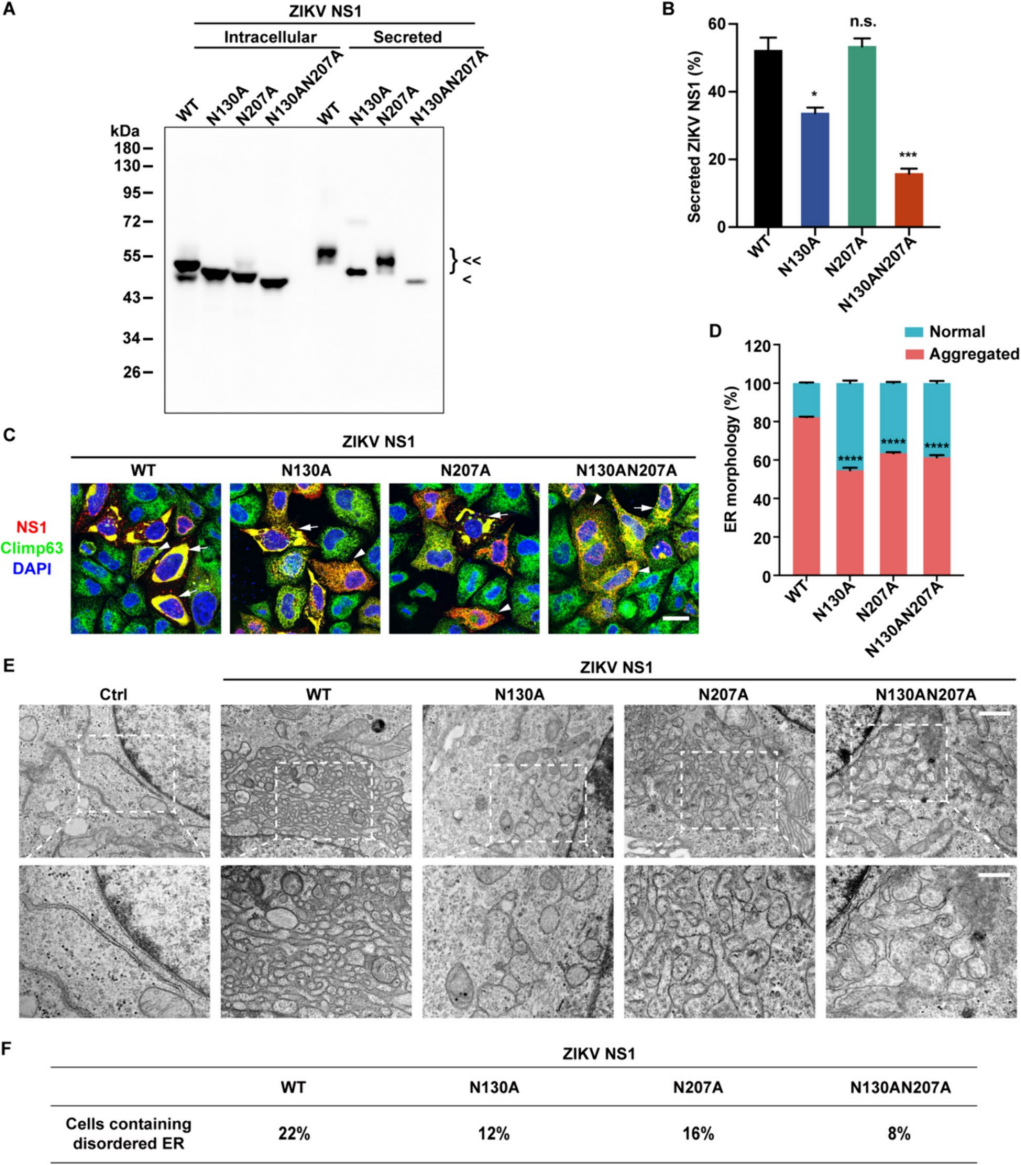
N-linked glycosylation of ZIKV NS1 is important for ER remodeling. **A** The glycosylation levels of WT and mutated ZIKV NS1. Intracellular and secreted ZIKV NS1 expressed in HeLa cells were analyzed by Western blotting. <  < , glycosylated NS1; < , non-glycosylated NS1(N130AN207A). **B** Quantification of secreted NS1. The percentage was calculated as secreted NS1 divided by the total NS1 (the sum of intracellular and secreted NS1). **C** NS1 induced ER morphology changes. HeLa cells expressing Myc-tagged WT or mutated ZIKV NS1 were stained with Myc and climp63 antibodies, with climp63 serving as the ER marker. NS1-positive cells were categorized into two groups based on ER morphology. “Aggregated ER” denotes ER aggregations above 5 μm^2^, as indicated by arrows; while “normal ER” indicates the ER maintaining sheet and tubule structures with ER aggregations below 5 μm^2^, as indicated by arrowheads. Scale bar, 20 μm. **D** Quantification of the cell population with different ER morphologies involved analyzing over 200 cells in each group. The percentage was calculated as the number of cells with aggregated ER divided by the total number of NS1-positive cells.** E** Ultrastructure of the ER in HeLa cells overexpressing WT or mutated ZIKV NS1 was captured by TEM. Ctrl, untransfected cells. Scale bar, 1 μm (upper), 500 nm (lower). **F** Quantification of TEM images in (**E**). The percentage of cells displaying specific ER morphologies among 50 cells was determined. All statistical data represent mean ± SEM (*n* = 3; *, *p* < 0.05; ***, *p* < 0.001; ****, *p* < 0.0001; n.s., non-significant; two-tailed t test)

**Fig. 2:**
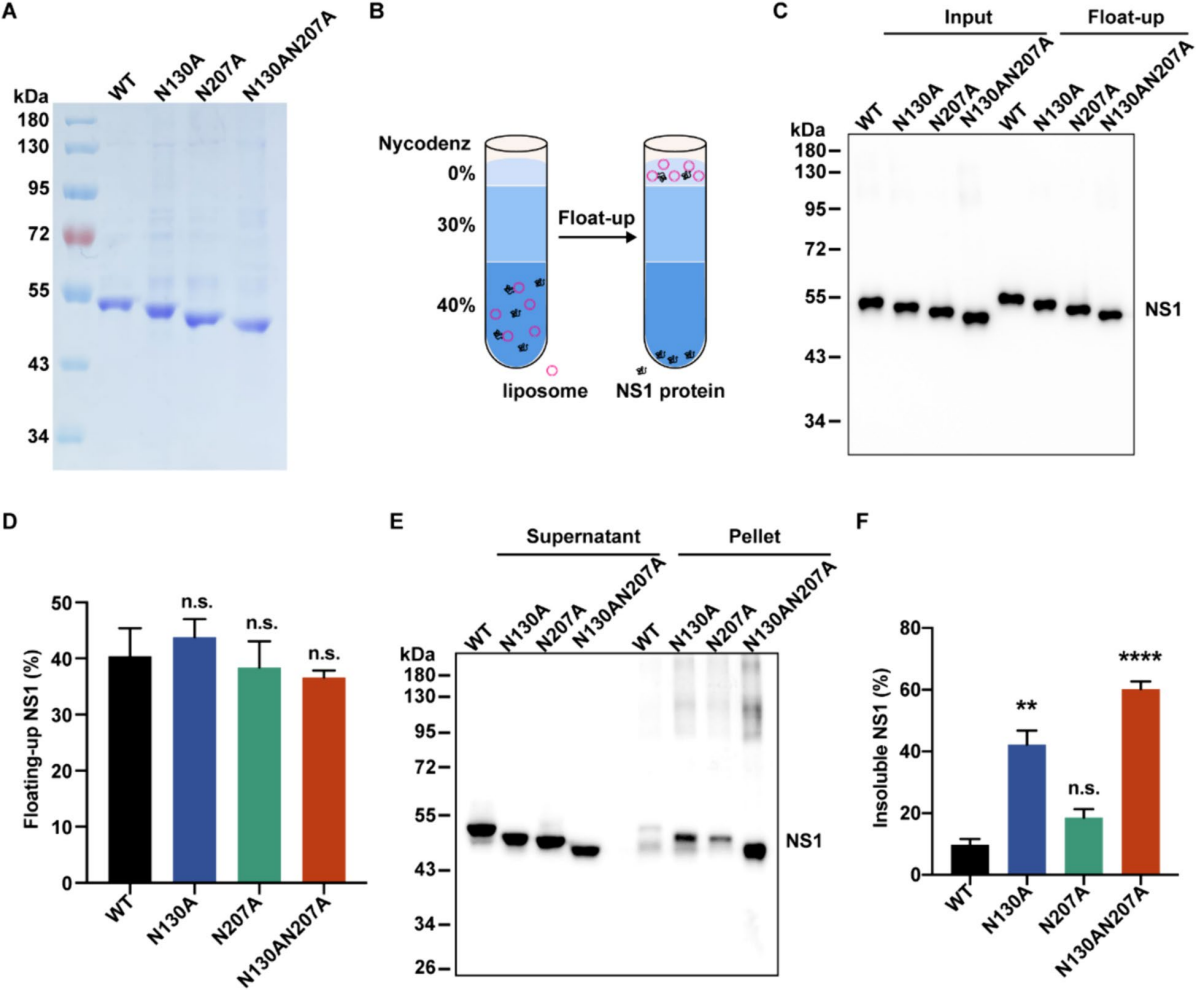
Glycosylation deficiency of ZIKV NS1 leads to protein aggregation. **A** The NS1 proteins with Twin-Strep-Tag, purified from HEK293T cells, were analyzed by Coomassie blue staining. **B** Schematic shows the liposome co-floating assay. NS1 proteins were incubated with liposomes, and then the mixture was applied to density gradient centrifugation. The proteins that floated up with liposomes were harvested from the top layer.** C** Co-floating assay of WT and mutated NS1. Input (2 times diluted with protein loading buffer) and float-up samples were analyzed by Western blotting. **D** Quantification of the co-floating NS1. Percentage was calculated as float-up NS1 divided by the input. **E** Glycosylation deficiency led to NS1 aggregation. HeLa cells were transfected with plasmids encoding for WT or mutated ZIKV NS1 proteins, and the soluble lysates and detergent-resistant pellets were analyzed by Western blotting. **F** Quantification of insoluble NS1 (detergent-resistant pellet) in (**E**). Percentage was calculated as insoluble NS1 divided by the total NS1 (the sum of soluble and insoluble NS1). All statistical data represent mean ± SEM (*n* = 3; **, *p* < 0.01; ****, *p* < 0.0001; n.s., non-significant; two-tailed t test)

**Fig. 3:**
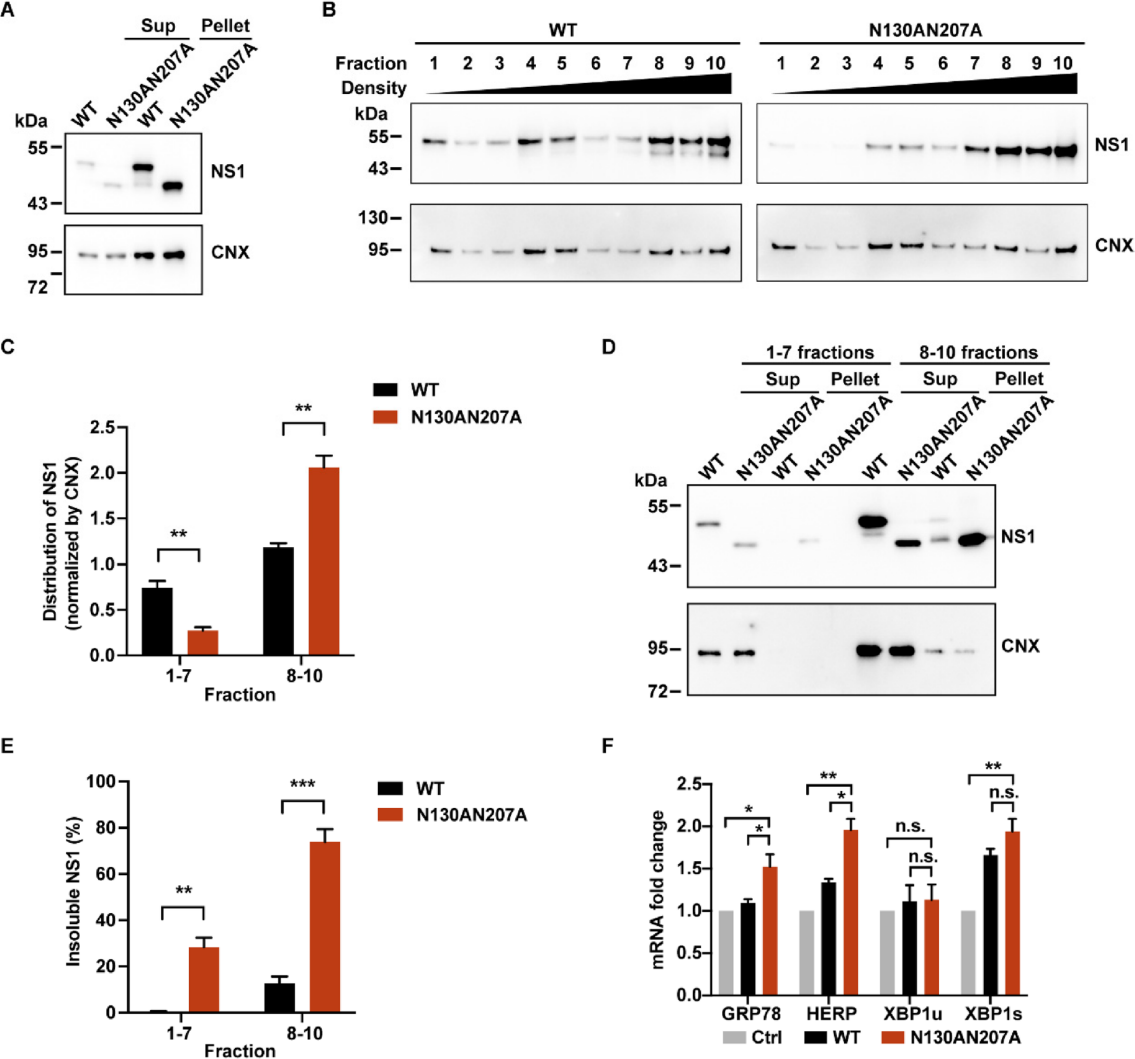
Glycosylation deficiency affects ER lumenal ZIKV NS1 solubility and ER association. **A** ZIKV NS1 and membrane-anchored CNX accumulated in pellet. HeLa cells expressing ZIKV NS1 proteins were subjected to ultrasonication and sequential centrifugations. The sup and pellet samples were analyzed by Western blotting. Sup, supernatant. CNX, calnexin.** B** The distributions of ZIKV NS1 and CNX in the pellets from (**A**) were separated by sucrose density gradient centrifugation. **C** The mutated NS1 was enriched in the high-density fractions. Distributions of ZIKV NS1 in fractions 1–7 and 8–10 were calculated as the amount of NS1 divided by that of CNX. **D** ZIKV NS1 N130AN207A mutant was accumulated in detergent-resistant pellet. Fractions 1–7 and 8–10 from (**B**) were harvested and treated with 1% NP-40, and the solubility was determined by Western blotting. **E** Quantification of insoluble NS1 (detergent-resistant pellet) in (**D**). The percentage was calculated as insoluble NS1 divided by the total NS1 (the sum of soluble and insoluble NS1). **F** N130AN207A mutant of ZIKV NS1 induced ER stress. Total RNA was extracted from HeLa cells and the mRNA levels of ER stress sensors were quantified by qPCR. Ctrl means untransfected cells. All statistical data represent mean ± SEM (*n* = 3; *, *p* < 0.05; **, *p* < 0.01; ***, *p* < 0.001; n.s., non-significant; two-tailed t test)

For Western blotting experiments, all samples were processed consistently. Equal numbers of cells were seeded in 24-well plates and lysed with the same volume of lysis buffer. Identical volumes of samples were then loaded onto the gels. In detail, for Figs. 1A and 5A, the cell medium was replaced with 300 μL Opti-MEM, and the cells were lysed with 100 μL lysis buffer. 6 μL of cell lysate and 18 μL of medium samples were loaded onto the gel. The percentage of secreted NS1 was determined by dividing secreted NS1 by the total NS1 (the sum of intracellular and secreted NS1). For Figs. 2E and 5H, the cells were lysed with 100 μL lysis buffer and centrifuged to separate the supernatant and pellet fractions. The pellet was resuspended with 100 μL 1 × SDS-PAGE loading buffer, and 10 μL of supernatant and pellet samples were loaded onto the gel. The percentage of insoluble NS1 was calculated by dividing insoluble NS1 in the pellet by the total NS1 (the sum of soluble and insoluble NS1). For Fig. 3A, the cells in 500 μL PBS were sonicated and centrifuged to pellet the membrane-associated proteins. The pellet was resuspended with 500 μL PBS, and 10 μL of supernatant and pellet samples were loaded onto the gel. For Coomassie blue staining, the total protein concentration was measured using a Nanodrop instrument (Thermo), and the NS1 protein was quantified with a BSA standard by SDS-PAGE. Therefore, for Fig. 2C, equal amount of NS1 proteins were loaded as input and the percentage of floating-up NS1 was calculated by dividing float-up NS1 by the input.

### Quantitative real-time PCR

RNA was purified with RNA fast200 (Fastagen, Cat. #220011) and qPCR was performed using FastKing one step Real-Time RT-PCR kit (TIANGEN, Cat. # FP313). Actin was used as the housekeeping gene. All primer sequences used in this study are shown below:

human GRP78 F: AAGGGGAACGTCTGATTGG

human GRP78 R: ACGGCAAGAACTTGATGTCC

human HERP F: GTCTCAGGGACTTGCTTCCA

human HERP R: TGTGGATTCAGCCACCTTGG

human Actin F: ATCGTCCACCGCAAATGCTTCTA

human Actin R: AGCCATGCCAATCTCATCTTGTT

ZIKV F: GGTCAGCGTCCTCTCTAATAAACG

ZIKV R: GCACCCTAGTGTCCACTTTTTCC

hamster Actin F: CAACTGGGACGATATGGAGAAG

hamster Actin R: TCTGGGTCATCTTTTCACGG

### Liposome co-floating assay

Liposome preparation and liposome co-floating assay were performed as previously described with some adjustments [21]. Briefly, 9 μL 150 mM DOPC (Avanti), 9.5 μL 15 mM cholesterol (Sigma-Aldrich) and 9.7 μL 0.77 mM Rhodamine-PE (Avanti) were mixed in 100 μL chloroform. The resulting lipid mixture was dried to a film using nitrogen gas and vacuumed for 2 h at RT to evaporate chloroform. The dried lipid mixture was dissolved with 800 μL solution buffer (50 mM Tris pH = 7.4, 100 mM NaCl) and vortexed for 30 min at RT. The dissolved mixture was subjected to seven freeze (liquid nitrogen)-and-thaw (water bath, 37 °C) cycles and extruded using Mini-Extruder (Avanti) through a 100 nm polycarbonate membrane filter (Whatman, 610,005). 1 μg of NS1 protein purified from HEK293T cells were incubated with 40 μL of 100 nm liposome at 37 °C for 2 h. Then the protein and liposome mixture were mixed with 80% Nycodenz to get 40% Nycodenz mixture. The Nycodenz gradient was prepared with 300 μL 40% Nycodenz containing the liposome and protein mixture, 250 μL 30% Nycodenz and 50 μL solution buffer from the bottom to the top. Samples were subjected to centrifugation at 45,000 rpm, 4 °C for 4 h. After centrifugation, proteins in the top layer were collected and analyzed by Western blotting.

### Analysis of detergent soluble and insoluble fraction

HeLa cells were transfected with NS1 plasmids. At 24 h post-transfection (hpt), the cells were rinsed with PBS and then lysed by RIPA buffer containing 1% NP-40 and protease inhibitors cocktail for 10 min on ice. The cell lysate was centrifuged for 10 min at 13,300 rpm, 4 °C, then the supernatant was transferred into a new tube. The supernatant and pellet samples were mixed with protein loading buffer, boiled for 10 min, and then submitted to Western blotting analysis.

### Sucrose density gradient centrifugation and fractionization

HeLa cells were transfected with WT or N130AN207A mutated ZIKV NS1 plasmids. At 24 hpt, cells were detached by scrapers, then centrifuged at 300 g, 4 °C for 5 min to harvest the cells. Cells were resuspended with PBS and subjected to ultrasonication for 5 s. Subsequently, the cell lysate was centrifuged at 700 g, 4 °C for 10 min to remove the nucleus and cell debris. The resulting supernatant was further centrifuged at 17,000 g, 4 °C for 30 min to isolate membrane components. The pellet obtained was resuspended in a 40% sucrose solution. Sucrose gradients were assembled by layering 1mL 50% sucrose, 1 mL 40% sucrose, 1 mL 40% sucrose containing the samples, and 1 mL 30% sucrose from bottom to top. The samples were subjected to ultracentrifuge for 6 h at 150,000 g, 4 °C with MLS-50 rotor (Beckman). Fractions were collected in 500 μL volumes from top to bottom. Fractions 1–7 and fractions 8–10 were mixed separately. Each 500 μL mixture (1–7 or 8–10) was then diluted into 5 mL PBS. The diluted mixtures were subjected to ultracentrifugation for 1 h at 150,000 g, 4 °C. The pellet obtained after ultracentrifugation was dissolved in RIPA buffer containing 1% NP-40 for 10 min on ice and subsequently centrifuged at 13,300 rpm, 4 °C for 10 min to extract the insoluble pellet.

### In vitro transcription

ZIKV replicon and ZIKV full length genome were transcribed in vitro using RiboMAXTM Large Scale RNA Production Systems-T7 (Promega, Cat. #P1300) and capped RNA was produced by the addition of Ribo m7G Cap Analog (Promega, Cat. #P1712). In vitro transcription was performed for 4 h at 37 °C. DNA template was removed by 2 μL RQ1 RNase-Free DNase for 30 min at 37 °C. The RNA products were purified by RNA fast200 (Fastagen) and stored at –80 °C in aliquots.

### Luciferase assay

ZIKV replicon WT clone was a gift from B. Zhang (Institute of Virology, Chinese Academy of Science, Wuhan, China). BHK-21 cells were transfected with ZIKV replicons (WT and mutants) carrying Renilla luciferase reporter by Viafect™ Transfect Reagent (Promega, Cat. #E4982). Luciferase activity of the ZIKV replicons was measured using Renilla Luciferase Assay System (Promega, Cat. #E2810) and GloMax® Navigator Microplate Luminometer at 10 h, 36 h and 48 h after transfection.

### Generation of ZIKV viruses and plaque assay

ZIKV generated by infectious DNA clone pFLZIKV was previously described [50]. Briefly, ZIKV full length genome RNA obtained from in vitro transcription was transfected into BHK-21 cells by Viafect transfection reagent. The culture media containing virus particles were collected when cytopathic effects occurred (about 72–96 hpt). The collected ZIKV stocks were tenfold diluted with DMEM without FBS. Vero cells were incubated with infection media for 2 h when the confluence reached 100%. Then the media were replaced by DMEM with 2% FBS containing 1.2% Methyl cellulose (Sigma, Cat. #M0512). After 5 days, Vero cells were fixed with 4% formaldehyde at RT for 1 h and stained with 1% crystal violet and visible plaques were counted.

### Quantification and statistical analysis

Secreted NS1 percentage:

$$secreted\, NS1 \left(\%\right)=\left(\frac{secreted\, NS1}{secreted \,NS1+intracellular \,NS1}\right)\times 100$$

This formula was applied for Fig. 1B, Fig. 5C, and Fig. S4C.

Glycosylated NS1 percentage:

$$glycosylated \,NS1 \left(\%\right)=\left(\frac{glycosylated\, NS1}{glycosylated \,NS1+non-glycosylated \,NS1}\right)\times 100$$

This formula was applied for Fig. 5B and Fig. S4B.

Insoluble NS1 percentage:

$$insoluble \,NS1 \left(\%\right)=\left(\frac{ NS1\, in \,pellet}{ NS1\, in\, sup+NS1 \,in \,pellet}\right)\times 100$$

This formula was applied for Fig. 2F, Fig. S2B.

Functional NS1 percentage:

$$functional \,NS1 \left(\%\right)=\left(\frac{ NS1\, in\, sup}{ NS1\, in\, sup+NS1 \,in \,pellet}\right)\times 100$$

This formula was applied for Fig. 5I and Fig. S4F.

Glycosylated insoluble NS1 percentage:

$$glycosylated\, insoluble NS1 \left(\%\right)=\left(\frac{glycosylated \,NS1 \,in \,\text{pellet}}{glycosylated \,NS1 \,in \,sup+glycosylated \,NS1 \,in \,\text{pellet}}\right)\times 100$$

Non-glycosylated insoluble NS1 percentage:

$$non-glycosylated\, insoluble \,NS1 \left(\%\right)=\left(\frac{non-glycosylated \,NS1\, in \,\text{pellet}}{non-glycosylated\, NS1\, in\, sup+non-glycosylated \,NS1\, in\, \text{pellet}}\right)\times 100$$

The above two formulas were applied for Fig. 5J and Fig. S4G.

All data in the results was performed three biological repeats and analyzed with Prism 8 software. The values represent mean ± SEM from three independent experiments and the statistical significance was determined by two-tailed t test. More than 200 cells were counted in the analysis of ER morphology. IC50 curve was fitted with [Inhibitor] vs. response – Variable slope (four parameters) in prism software.

## Results

### N-linked glycosylation is important for ER remodeling of NS1

To assess the impact of NS1 glycosylation, we constructed three N-linked glycosylation mutants for ZIKV and DENV NS1, comprising two single mutants (N130A and N207A) and a double mutant (N130AN207A). Due to distinct glycosylation modifications, wild type (WT) and mutated NS1 from HeLa cells exhibited different molecular weights in Western blotting. Both ZIKV NS1 single mutants (N130A and N207A) displayed slightly lower molecular weights than WT, indicating hypo-glycosylation (partial glycosylation). The double mutant (N130AN207A) had the lowest molecular weight due to non-glycosylation (Fig. 1A). We further scrutinized the glycosylation of secreted NS1, considering its role as a secreted protein. Secreted WT NS1 and NS1 N207A proteins exhibited larger molecular weights compared to their intracellular counterparts, indicating further glycosylation during the secretion pathway. Nonetheless, secreted NS1 mutants had lower molecular weights than secreted WT NS1, indicating glycosylation deficiency (Fig. 1A). Moreover, the N130A and N130AN207A mutations in ZIKV NS1 dramatically reduced NS1 secretion, whereas the N207 mutation had no significant effect (Fig. 1A and B). Similarly, all three DENV NS1 mutants exhibited lower molecular weights due to glycosylation deficiency (Fig. S1A). For DENV NS1, both N130- and N207- glycosylation depletions dramatically reduced NS1 secretion, and all the mutants can hardly be detected in secretory media (Fig. S1A) [26, 31]. These data affirm that N130 and N207 are the main glycosylation sites of flavivirus NS1, and N-linked glycosylation profoundly influences NS1 secretion.

Considering NS1’s involvement in the formation of viral RCs through ER remodeling, we explored the impact of NS1 glycosylation on ER reorganization. As previously demonstrated, overexpression of WT ZIKV NS1 in the ER lumen induced apparent ER aggregation [21]. However, glycosylation deficiency of ZIKV NS1 attenuated its ER remodeling activity, as evidenced by mutations (N130A, N207A, and N130AN207A) that reduced the percentage of cells with aggregated ER (Fig. 1C and D). In comparison to ZIKV NS1, DENV NS1 exhibited weaker ER remodeling activity. Still, the double mutation (N130AN207A) of DENV NS1 impaired its ER remodeling ability (Fig. S1B and S1C). Further examination of ER ultrastructure via transmission electron microscope (TEM) revealed aggregated small vesicular structures resembling viral RCs in WT NS1-expressing cells (Fig. 1E and S1D). However, such structures were less frequent and larger in cells expressing NS1 mutants (Fig. 1E, F, and S1D). Consequently, glycosylation emerges as a critical factor for NS1’s ER remodeling activity.

### Glycosylation deficiency does not affect membrane binding activity of NS1 but leads to abnormal aggregation and inactivation of NS1

Since NS1 remodels ER depending on its association with the lipid membrane, we hypothesized that the effect of NS1 glycosylation deficiency on ER remodeling might arise from interfering with its association with the lipid membrane. To investigate this, we performed a liposome co-floating assay in vitro. WT ZIKV NS1 and three mutants with C-terminal Twin-Strep-Tag were expressed in HEK293T cells and purified by Streptactin beads (Fig. 2A). The NS1 proteins were incubated with liposomes and subjected to density gradient centrifugation. After ultracentrifugation, the top layer contained liposomes and co-floated proteins that associates with liposomes (Fig. 2B). We found that both WT and mutated NS1 bound to the liposomes and co-floated. Surprisingly, there is no significant difference in the co-floating assay between the amounts of WT NS1 and mutants (Fig. 2C and D), suggesting that glycosylation deficiency does not affect the membrane binding activity of NS1. Given the conformation of the NS1 dimer, with N130 and N207 located on the face far away from the lipid membrane, it is reasonable to conclude that glycosylation may not affect NS1’s association with the membrane.

However, the question remains: how could glycosylation deficiency affect NS1’s ER remodeling activity? We noticed that N-glycosylation site mutations significantly reduced NS1 secretion, and these NS1 mutants exhibited lower protein yield compared with WT NS1 during purification. Therefore, we postulated that hypo- and non-glycosylated NS1 mutants might accumulate in insoluble components. To test this hypothesis, we centrifuged cell lysates and examined NS1 amounts in soluble supernatants and insoluble pellets. Results indicated that N130 and N207 single mutations led to approximately 40% and 18%, respectively, of mutated NS1 accumulating in insoluble pellets, compared with less than 10% of WT NS1 (Fig. 2E and F). In contrast, over 60% of NS1 double mutant (N130AN207A) appeared in insoluble pellet, suggesting that non-glycosylation dramatically induced NS1 aggregation and inactivation (Fig. 2E and F). The N207 mutation appeared to have a milder effect on protein solubility compared with the N130 mutation and double mutations (Fig. 2E and F). Similarly, glycosylation deficiency also affected DENV NS1 solubility (Fig. S2A and S2B). Additionally, we observed that a much higher percentage of WT DENV NS1 was insoluble (close to 40%) compared with WT ZIKV NS1 (less than 10%) (Fig. 2E, F, S2A, and S2B), suggesting varying solubility among flavivirus NS1 proteins. This difference might explain why the ER remodeling activity of DENV NS1 was weaker than that of ZIKV NS1 (Fig. 1D and S1C), owing to less soluble and functional DENV NS1 existing in the ER lumen.

### Glycosylation deficiency affects ER lumenal NS1 solubility and ER association

To further identify the status of NS1 in the cells, we subjected cells to ultrasonication (without detergent) followed by sequential centrifugations (low-speed centrifugation of 700 g for 10 min, then high-speed centrifugation of 17,000 g for 30min). Given that flavivirus NS1 is localized in the ER lumen and binds to ER membrane, we first examined whether NS1 existed in the ER fraction. Calnexin (CNX), an integral ER membrane protein, served as an ER marker. Since no detergent was used during ultrasonication, only soluble proteins would appear in supernatants after high-speed centrifugation. Most CNX was found in the pellets after high-speed centrifugation, suggesting that the majority of cellular ER fractions were concentrated in the pellets (Fig. 3A). Similarly, the majority of NS1 was enriched in the pellets (Fig. 3A).

Next, we resuspended the pellets and performed fractionation by sucrose density gradient centrifugation. The comparable distributions between ZIKV NS1 and CNX indicated that WT NS1 is closely associated with the ER membrane (Fig. 3B and C). However, a substantial proportion of non-glycosylated NS1 (N130AN207A) shifted to the high-density fractions (fractions 8–10), which suggests functional changes in non-glycosylated NS1 (Fig. 3B and C). To examine the solubility of NS1 in low- (fractions 1–7) and high-density (fractions 8–10) fractions, we collected the components from both sets of fractions by centrifugation. We then resuspended the pellets with a buffer containing 1% NP-40 and centrifuged the samples again, analyzing the supernatants and pellets by Western blotting. The data indicated that non-glycosylation resulted in significant insolubility of NS1 (N130AN207A), particularly in the high-density fractions 8–10. Meanwhile, most CNX was soluble and present in supernatants (Fig. 3D and E).

Both the shift in sucrose fractionation and the higher insolubility of N130AN207A mutant suggested the dysfunction of non-glycosylated NS1. Considering the abnormal aggregation of NS1 in the ER, we hypothesized that ER stress might be activated. To examine ER stress, we quantified the mRNA changes in several ER stress markers, including GRP78, HERP, and XBP1. ZIKV NS1 N130AN207A mutant induced a higher level of ER stress response compared with WT ZIKV NS1 (Fig. 3F). For DENV NS1, non-glycosylation did not induce stronger ER stress response, possibly due to the relative higher ER stress response induced by WT DENV NS1, which inherently has high insolubility (Fig. S2C). In conclusion, we demonstrated that the abnormal aggregation of non-glycosylated NS1 contributes to the attenuation of ER remodeling and the induction of more ER stress.

### N-linked glycosylation of NS1 ensures efficient virus replication

**Fig. 4:**
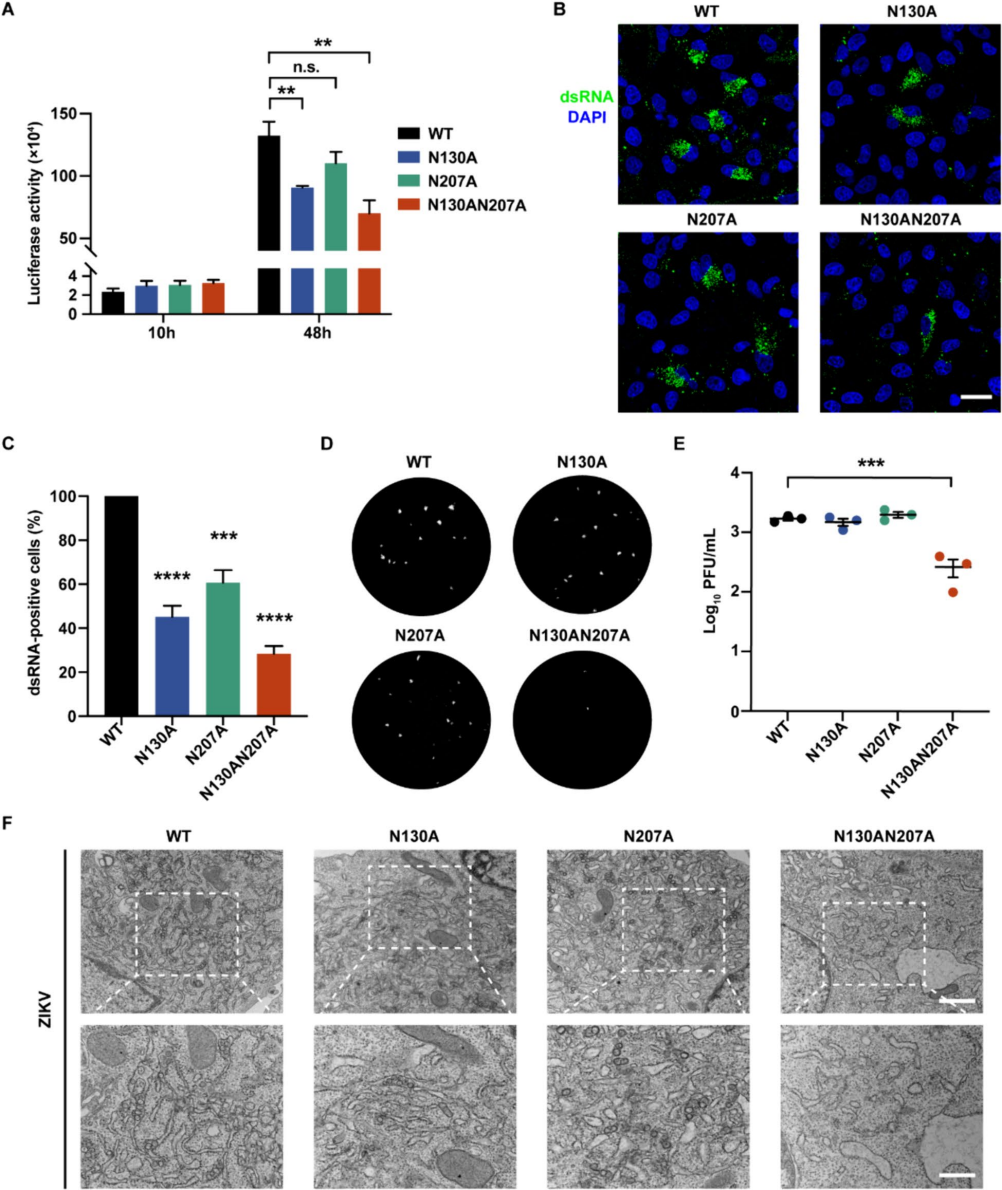
N-linked glycosylation of ZIKV NS1 ensures effective virus replication. **A** Renilla luciferase assay of ZIKV replicons. The luciferase activity was measured at 10 h and 48 h after transfection of BHK-21 cells with WT or mutated ZIKV replicon RNA. **B** Immunofluorescence assay detecting the replication efficiency. BHK-21 cells transfected with ZIKV replicons were stained with a dsRNA antibody (Green: dsRNA, Blue: DAPI). Scale bar, 20 μm. **C** Quantification of dsRNA-positive cells involved analyzing 20 views for each group. WT ZIKV replicon is set as 100%. **D** Plaque assays detecting infectious ZIKV production were performed in Vero cells on day 5 after infection. **E** The number of plaques per mL was counted and analyzed statistically. **F** Ultrastructure of the ER in BHK-21 cells infected with WT or mutated ZIKV was captured by TEM. Scale bar, 1 μm (upper), 500 nm (lower). All statistical data represent mean ± SEM (*n* = 3; **, *p* < 0.01; ***, *p* < 0.001; ****, *p* < 0.0001; n.s., non-significant; two-tailed t test)

To determine the impact of NS1 glycosylation on virus replication, we introduced N130A, N207A, and N130AN207A mutations into ZIKV replicon. The ZIKV replicon contains the complete ZIKV virus genome sequence, but with the viral structural genes replaced by a luciferase gene as a reporter [33]. Therefore, replication efficiency of WT and mutated ZIKV replicons can be indicated by the luciferase activity. In comparison to the WT replicon, all three mutated replicons exhibited decreased luciferase activity (Fig. 4A). Immunostaining of dsRNA (viral double-stranded RNA intermediates during replication) also confirmed that N-linked glycosylation mutations of NS1 attenuated viral RNA synthesis (Fig. 4B and C).

Furthermore, we constructed ZIKV infectious clones carrying NS1 mutations (ZIKV-FL-N130A, ZIKV-FL-N207A, and ZIKV-FL-N130AN207A). We transfected BHK-21 cells with these infectious clones and determined viral titers in the cells culture media using plaque assay. ZIKV-FL-N130AN207A produced fewer and smaller plaques, suggesting an attenuated virus yield (Fig. 4D and E). Through TEM images, we identified that ZIKV with non-glycosylated NS1 (N130AN207A) inhibited viral RCs formation in BHK-21 cells and HeLa cells (Fig. 4F and S3). Collectively, our results indicated that N-linked glycosylation of NS1 plays an important role in ensuring efficient flavivirus replication.

### Tunicamycin blocks NS1 glycosylation and interferes with its ER remodeling

**Fig. 5:**
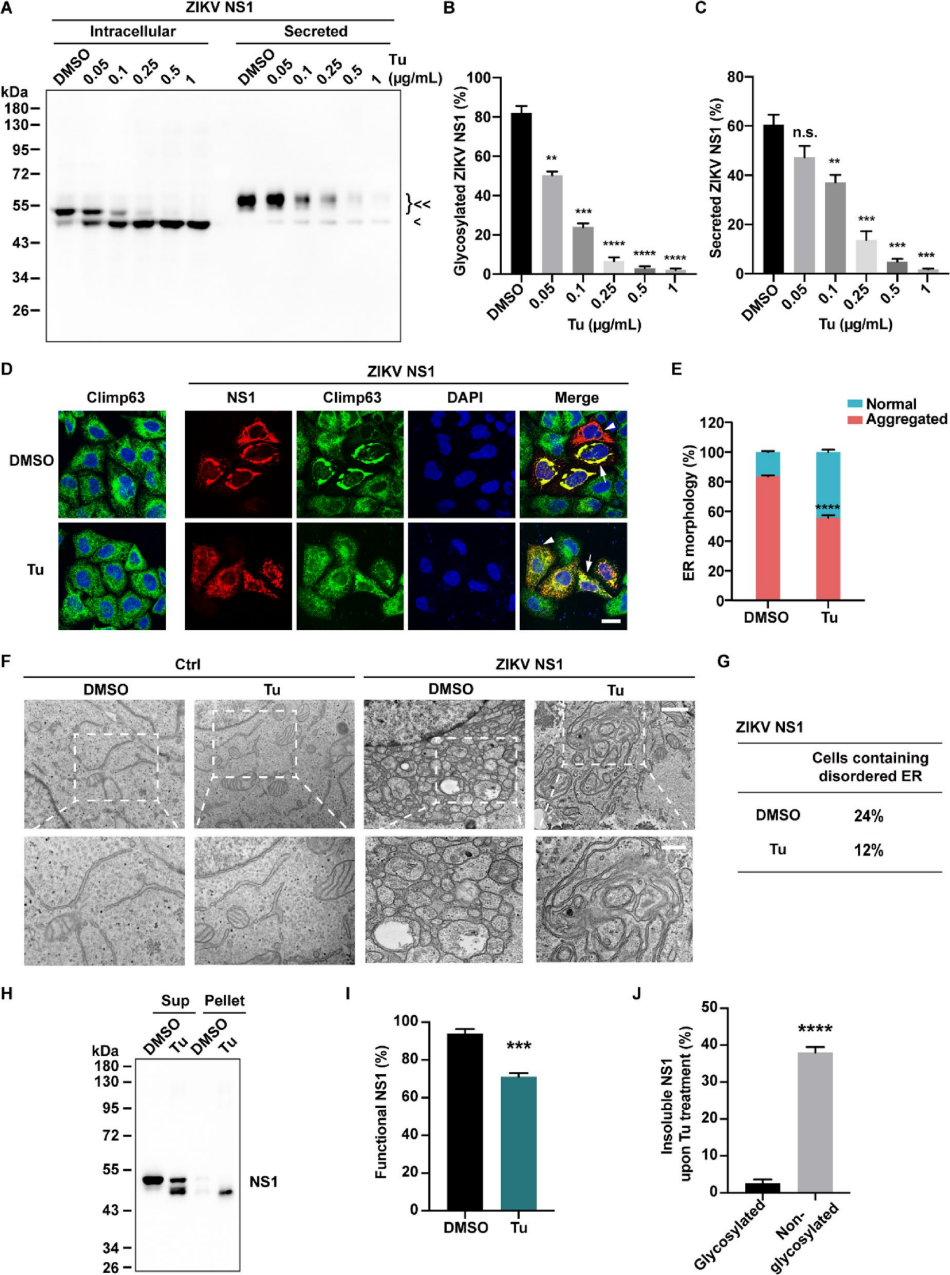
Tunicamycin blocks ZIKV NS1 glycosylation and interferes with its ER remodeling. **A** The glycosylation levels of ZIKV NS1 WT under tunicamycin (Tu) treatment. Intracellular and secreted NS1 proteins expressed in HeLa cells were immunoblotted with the Myc antibody. <  < , glycosylated NS1; < , non-glycosylated NS1. DMSO as a control. **B** Quantification of glycosylated NS1. The percentage was calculated as glycosylated NS1 divided by the total intracellular NS1. **C** Quantification of secreted NS1. The percentage was calculated as secreted NS1 divided by the total NS1 (the sum of intracellular and secreted NS1). **D** ZIKV NS1 induced ER morphology changes with Tu treatment. HeLa cells were treated with 0.5 μg/mL Tu or transfected with Myc-tagged WT NS1 plasmid, followed by treatment with 0.5 μg/mL Tu. Cells were fixed and stained with Myc and climp63 antibodies. Arrows indicate cells with aggregated ER, arrowheads indicate cells with normal ER. Scale bar, 20 μm. **E** Quantification of the cell population with different ER morphologies. The percentage was calculated as the number of cells with aggregated ER divided by the total number of NS1-positive cells. **F** TEM images showed the ER ultrastructure in HeLa cells treated with 0.25 μg/mL Tu or transfected with WT ZIKV NS1 plasmid and treated with 0.25 μg/mL Tu. Scale bar, 1 μm (upper), 500 nm (lower). **G** Quantification of TEM images in (**F**). The percentage of cells displaying specific ER morphologies upon transfection with the ZIKV NS1 plasmid among 50 cells was determined and listed in the table. **H** Tu induced ZIKV NS1 aggregation. HeLa cells were transfected with WT ZIKV NS1 plasmid and treated with 0.5 μg/mL Tu. Then the soluble supernatants and detergent-resistant pellets were analyzed by Western blotting. **I** Quantification of functional NS1 (detergent-soluble supernatant) in (**H**). The percentage was calculated as soluble NS1 in the supernatant divided by the total NS1 (the sum of soluble and insoluble NS1). **J** Quantification of glycosylated or non-glycosylated insoluble NS1 upon Tu treatment in (**H**). The percentage of insoluble NS1 was calculated by dividing glycosylated or non-glycosylated insoluble NS1 by the total glycosylated or non-glycosylated NS1, respectively. All statistical data represent mean ± SEM (*n* = 3; **, *p* < 0.01; ***, *p* < 0.001; ****, *p* < 0.0001; n.s., non-significant; two-tailed t test)

As flavivirus NS1 is glycosylated in the ER, and its glycosylation affects virus replication, we attempted to block NS1 glycosylation using tunicamycin, an inhibitor of N-linked glycosylation by blocking the transfer of UDP-N-acetylglucosamine (GlcNAc) to dolichol phosphate in the ER of eukaryotic cells [34]. Tunicamycin treatment markedly decreased both the molecular weights and secretion of NS1 proteins in a dose-dependent manner, confirming the effective inhibition of glycosylation in both ZIKV and DENV NS1 (Fig. 5A-C and S4A-C).

Next, we examined the ER remodeling ability of NS1 in the presence of tunicamycin. As anticipated, tunicamycin itself did not affect ER morphology but interfered with ZIKV NS1-induced ER remodeling (Fig. 5D and E). Moreover, TEM images confirmed that tunicamycin treatment significantly altered the ultrastructure of ER remodeling induced by ZIKV or DENV NS1, exhibiting less dense ER aggregation and fewer small vesicular structures (Fig. 5F, G and S4D). Consistent with the abovementioned results, both ZIKV and DENV NS1 accumulated in the pellet fraction, indicating reduced functional NS1 levels following tunicamycin treatment (Fig. 5H, I, S4E and S4F). Moreover, non-glycosylated NS1 is more instable and tends to accumulate in pellet (Fig. 5J and S4G). Therefore, tunicamycin treatment induced non-glycosylation of flavivirus NS1, thereby impairing its ER remodeling activity by decreasing the solubility of NS1.

### Tunicamycin inhibits flavivirus replication

**Fig. 6:**
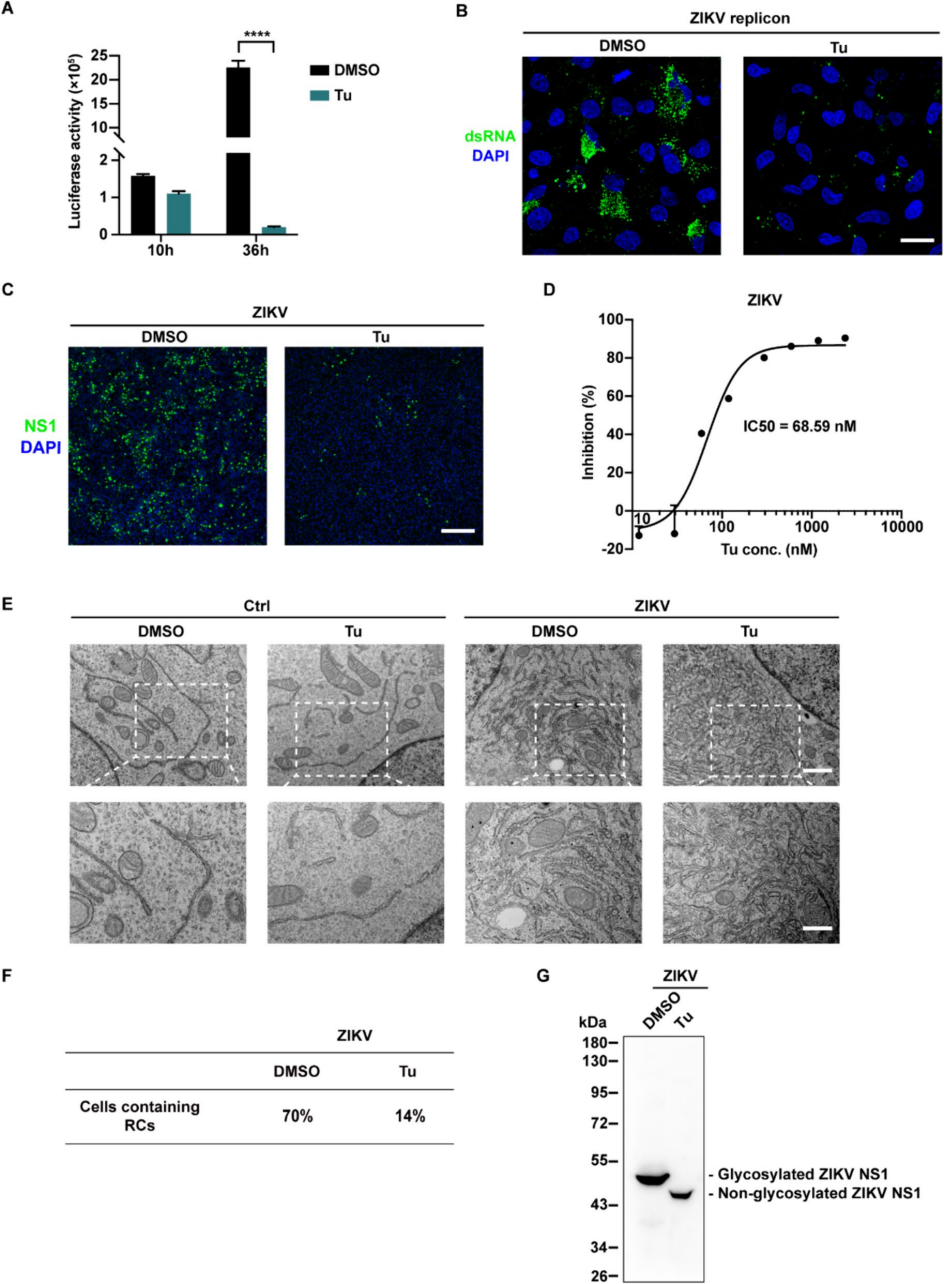
Tunicamycin inhibits ZIKV replication. **A** Renilla luciferase assay of ZIKV replicons. The luciferase activity was measured at 10 h and 36 h after the transfection of BHK-21 cells with WT ZIKV replicon RNA. Cells were treated with Tu (0.5 μg/mL) at 10 h post-transfection. **B** Immunofluorescence assay detecting replication efficiency. BHK-21 cells transfected with WT ZIKV replicon were treated with Tu (0.5 μg/mL) and stained with a dsRNA antibody (Green: dsRNA, Blue: DAPI). Scale bar, 20 μm. **C** Tu inhibited ZIKV replication. ZIKV-infected BHK-21 cells were treated with Tu (0.25 μg/mL) at 8 h post-infection (hpi) and stained with NS1 antibody at 24 hpi. Scale bar, 100 μm. **D** IC50 curve of Tu for ZIKV replication. ZIKV replication levels in BHK-21 cells under Tu treatment were quantified by qPCR assay at 24 hpi. The IC50 value is 68.59 nM. The statistical data represent mean ± SEM. **E** Tu inhibited ZIKV RCs formation. TEM images showed the ER ultrastructure in BHK-21 cells treated with 0.25 μg/mL Tu or infected with ZIKV and treated with 0.25 μg/mL Tu. Scale bar, 1 μm (upper), 500 nm (lower). **F** Quantification of TEM images in (**E**). The percentage of cells containing RCs upon infection with ZIKV among 50 cells was determined. **G** The glycosylation status of ZIKV NS1 in ZIKV-infected BHK-21 cells treated with Tu was detected by Western blotting with NS1 antibody at 24 hpi

To explore the effectiveness of tunicamycin in inhibiting ZIKV replication, we conducted a ZIKV replicon luciferase assay and performed immunostaining against viral dsRNA. As shown in Fig. 6A and B, tunicamycin markedly inhibited ZIKV replicon replication. When BHK-21 cells were infected with ZIKV or DENV, tunicamycin significantly reduced virus replication (Fig. 6C and S5A). The IC50 (half-maximal inhibition concentration) for tunicamycin against ZIKV and DENV were 68.59 nM and 78.91 nM, respectively (Fig. 6D and S5B). Furthermore, tunicamycin treatment resulted in the deficiency of viral RCs in infected cells, suggesting that tunicamycin inhibited viral replication by disrupting the formation of RCs (Fig. 6E, F and S5C). As anticipated, we found that tunicamycin blocked NS1 glycosylation in ZIKV- and DENV-infected cells (Fig. 6G and S5D). These findings unveiled the underlying mechanism by which tunicamycin inhibits flavivirus replication and validated the pivotal role of NS1 glycosylation in flavivirus RCs formation. Thus, tunicamycin might inhibit flavivirus replication by impeding the NS1 glycosylation process. By consolidating the findings from NS1 glycosylation mutants, we demonstrated the crucial role of NS1 glycosylation in flavivirus RCs formation.

## Discussion

Glycosylation is a critical post-translational modification influencing protein folding, stability, recognition, and various cellular functions [35, 36]. Previous studies had emphasized the significance of NS1 glycosylation in flaviviral replication and virulence [30, 32, 37, 38]. This importance is underscored by the development of vaccine platforms incorporating NS1 glycosylation mutations for generating live attenuated ZIKV vaccines, with subsequent evaluations demonstrating protective effects in mice [32, 39]. A Study have shown that PDK53, a dengue virus candidate vaccine, causes ER stress by impacting NS1 glycosylation, thereby reducing virus infection [38]. Our findings align with previous studies, confirming that ZIKV NS1 glycosylation is important for effective virus replication. While these outcomes strongly suggest a crucial role for glycosylation in NS1 function and viral replication, the specific mechanism underlying this influence remains unclear.

Earlier work by Pryor et al. revealed that the absence of N207-glycosylation in DENV-2 NS1 showed reduced dimer stability and lower secretion level [40]. Similarly, Somnuke et al. indicated that N-linked glycosylation influences secretion yield and soluble NS1 stability [26]. Our study expands on these observations by demonstrating that NS1 glycosylation deficiency leads to abnormal aggregation in the ER. This implies that NS1 glycosylation aids in stabilizing NS1 proteins and enhance their solubility in the ER, the site of virus replicates. Consequently, the intracellular NS1 aggregation resulting from glycosylation deficiency provides an explanation for the reduced protein stability and lower secretion levels observed in earlier reports.

Structural studies have revealed the presence of secreted flaviviral NS1 in dimeric, tetrameric, and hexameric states, with tetrameric or hexameric NS1 formed by two or three dimeric NS1 units. However, the function and mechanism of NS1 oligomer formation remain subjects of ongoing discussion [28, 41]. Moreover, evidence has indicated the existence of higher-order oligomers (> 675 kDa) in secreted NS1, and glycosylation mutations were found to increase the prevalence of these oligomers [20, 26]. Notably, this aligns with our findings that non-glycosylation of flavivirus NS1 tends to form high-density aggregates within cells. Hence, it is reasonable to posit that glycosylation not only determines the stability of NS1 but also governs its various oligomeric states because higher-order oligomeric NS1 may be more prone to form aggregation.

While prior studies provided clues into the role of glycosylation in NS1 function, they did not establish a direct relationship between NS1 glycosylation and viral replication [26, 28, 37, 40]. Our recent work demonstrated the essential role of flavivirus NS1 in viral replication, particularly in the formation of RCs [21]. In the present study, our data showed that the glycosylation modifications of flavivirus NS1 are important for RCs formation. Glycosylation deficiency caused NS1 aggregation in the ER, resulting in the decrease of functional NS1 and impairment of its ER remodeling activity. Consequently, viral RCs formation was affected, leading to the inhibition of virus replication. Thus, our findings directly address how NS1 glycosylation affects flavivirus replication.

Viral RCs serve as the platform for efficient virus replication, making them an ideal target for intervention [15, 42]. We found that tunicamycin, by blocking NS1 glycosylation, effectively inhibited ZIKV and DENV replication. Notably, tunicamycin exhibited a stronger inhibitory effect than NS1 N-glycosylation mutations. This difference can be attributed to several factors. Firstly, tunicamycin treatment induces ER stress, which has been shown to synergize with pattern recognition sensing to inhibit viral replication during flavivirus infection [43, 44]. The treatment of tunicamycin might induce robust ER stress, enhancing ER stress-related antiviral properties. Secondly, tunicamycin not only blocks NS1 glycosylation but also affects the glycosylation of other viral and host proteins [45]. It’s known that tunicamycin blocks glycosylation of viral envelope proteins, as demonstrated in studies involving ZIKV and hepatitis C virus [46, 47]. Additionally, tunicamycin has been proposed as a potential anti-coronavirus drug by acting on the S protein [48, 49]. These instances illustrate that tunicamycin interferes with virus infection at the entry step by blocking the glycosylation of viral envelope proteins. However, few studies provide evidence that tunicamycin directly acts on viral replication. Our study unravels a novel mechanism wherein tunicamycin directly inhibits flavivirus replication by blocking NS1 protein glycosylation and interfering with viral RCs formation. Given that a variety of positive-sense RNA viruses replicate in membranous RCs, tunicamycin may affect the formation of multiple viral RCs, inhibiting the replication of various viruses. Thus, tunicamycin holds promise as a potential antiviral drug against multiple viruses, including flaviviruses, and further studies need to be investigated.

## Conclusions

We found that the glycosylation deficiency of NS1, either through N-glycosylation site mutations or tunicamycin (an inhibitor of N-linked glycosylation) treatment, compromises its ER remodeling activity and interferes with viral RCs formation, resulting in the inhibition of flavivirus replication. Glycosylation deficiency results in abnormal aggregation of NS1 in the ER, rather than reducing its membrane-binding activity. In summary, our work reports the function of glycosylation on NS1 involved in RCs formation, providing a new anti-flavivirus strategy.

### Supplementary Information

Supplementary Material 1.

Supplementary Material 2.

Supplementary Material 3.

## Data Availability

Not applicable.

## References

[1] Mukhopadhyay S, Kuhn RJ, Rossmann MG (2005). A structural perspective of the flavivirus life cycle. Nat Rev Microbiol.

[2] Musso D, Gubler DJ (2016). Zika Virus. Clin Microbiol Rev.

[3] Roy SK, Bhattacharjee S (2021). Dengue virus: epidemiology, biology, and disease aetiology. Can J Microbiol.

[4] Pierson TC, Diamond MS (2020). The continued threat of emerging flaviviruses. Nat Microbiol.

[5] Plourde AR, Bloch EM (2016). A Literature Review of Zika Virus. Emerg Infect Dis.

[6] Bhatt S (2013). The global distribution and burden of dengue. Nature.

[7] Guzman MG, Harris E (2015). Dengue. Lancet.

[8] Kularatne SA (2015). Dengue fever. BMJ.

[9] Barrett ADT (2018). Current status of Zika vaccine development: Zika vaccines advance into clinical evaluation. NPJ Vaccines.

[10] Wilder-Smith A (2019). Deliberations of the Strategic Advisory Group of Experts on Immunization on the use of CYD-TDV dengue vaccine. Lancet Infect Dis.

[11] Troost B, Smit JM (2020). Recent advances in antiviral drug development towards dengue virus. Curr Opin Virol.

[12] Dalrymple N.A, Cimica V, Mackow E.R (2015). Dengue Virus NS Proteins Inhibit RIG-I/MAVS Signaling by Blocking TBK1/IRF3 Phosphorylation: Dengue Virus Serotype 1 NS4A Is a Unique Interferon-Regulating Virulence Determinant. mBio.

[13] Xia H (2018). An evolutionary NS1 mutation enhances Zika virus evasion of host interferon induction. Nat Commun.

[14] Bollati M (2010). Structure and functionality in flavivirus NS-proteins: perspectives for drug design. Antiviral Res.

[15] Ci Y, Shi L (2021). Compartmentalized replication organelle of flavivirus at the ER and the factors involved. Cell Mol Life Sci.

[16] Welsch S (2009). Composition and three-dimensional architecture of the dengue virus replication and assembly sites. Cell Host Microbe.

[17] Klaitong P, Smith DR (2021). Roles of Non-Structural Protein 4A in Flavivirus Infection. Viruses.

[18] Miller S (2007). The non-structural protein 4A of dengue virus is an integral membrane protein inducing membrane alterations in a 2K-regulated manner. J Biol Chem.

[19] Wang Y, Xie X, Shi PY (2022). Flavivirus NS4B protein: Structure, function, and antiviral discovery. Antiviral Res.

[20] Youn S (2012). Evidence for a genetic and physical interaction between nonstructural proteins NS1 and NS4B that modulates replication of West Nile virus. J Virol.

[21] Ci Y (2020). Zika NS1-induced ER remodeling is essential for viral replication. J Cell Biol.

[22] Rastogi M, Sharma N, Singh SK (2016). Flavivirus NS1: a multifaceted enigmatic viral protein. Virol J.

[23] Flamand M (1999). Dengue virus type 1 nonstructural glycoprotein NS1 is secreted from mammalian cells as a soluble hexamer in a glycosylation-dependent fashion. J Virol.

[24] Shu B (2022). CryoEM structures of the multimeric secreted NS1, a major factor for dengue hemorrhagic fever. Nat Commun.

[25] Xu X (2016). Contribution of intertwined loop to membrane association revealed by Zika virus full-length NS1 structure. EMBO J.

[26] Somnuke P (2011). N-linked glycosylation of dengue virus NS1 protein modulates secretion, cell-surface expression, hexamer stability, and interactions with human complement. Virology.

[27] Akey DL (2015). Structure-guided insights on the role of NS1 in flavivirus infection. BioEssays.

[28] Yap SSL (2017). Dengue Virus Glycosylation: What Do We Know?. Front Microbiol.

[29] Tajima S, Takasaki T, Kurane I (2008). Characterization of Asn130-to-Ala mutant of dengue type 1 virus NS1 protein. Virus Genes.

[30] Pryor MJ (1998). Growth restriction of dengue virus type 2 by site-specific mutagenesis of virus-encoded glycoproteins. J Gen Virol.

[31] Crabtree MB, Kinney RM, Miller BR (2005). Deglycosylation of the NS1 protein of dengue 2 virus, strain 16681: construction and characterization of mutant viruses. Arch Virol.

[32] Richner JM (2017). Vaccine Mediated Protection Against Zika Virus-Induced Congenital Disease. Cell.

[33] Li JQ (2018). Development of a replicon cell line-based high throughput antiviral assay for screening inhibitors of Zika virus. Antiviral Res.

[34] Surani MA (1979). Glycoprotein synthesis and inhibition of glycosylation by tunicamycin in preimplantation mouse embryos: compaction and trophoblast adhesion. Cell.

[35] Very N, Lefebvre T, El Yazidi-Belkoura I (2018). Drug resistance related to aberrant glycosylation in colorectal cancer. Oncotarget.

[36] Reily C (2019). Glycosylation in health and disease. Nat Rev Nephrol.

[37] Muylaert IR (1996). Mutagenesis of the N-linked glycosylation sites of the yellow fever virus NS1 protein: effects on virus replication and mouse neurovirulence. Virology.

[38] Choy MM (2020). A Non-structural 1 Protein G53D Substitution Attenuates a Clinically Tested Live Dengue Vaccine. Cell Rep.

[39] Annamalai AS (2019). An Attenuated Zika Virus Encoding Non-Glycosylated Envelope (E) and Non-Structural Protein 1 (NS1) Confers Complete Protection against Lethal Challenge in a Mouse Model. Vaccines (Basel).

[40] Pryor MJ, Wright PJ (1994). Glycosylation mutants of dengue virus NS1 protein. J Gen Virol.

[41] Muller DA, Young PR (2013). The flavivirus NS1 protein: molecular and structural biology, immunology, role in pathogenesis and application as a diagnostic biomarker. Antiviral Res.

[42] van den Elsen K, Quek JP, Luo D (2021). Molecular Insights into the Flavivirus Replication Complex. Viruses.

[43] Carletti T (2019). Viral priming of cell intrinsic innate antiviral signaling by the unfolded protein response. Nat Commun.

[44] Mufrrih M, Chen B, Chan SW (2021). Zika Virus Induces an Atypical Tripartite Unfolded Protein Response with Sustained Sensor and Transient Effector Activation and a Blunted BiP Response. mSphere.

[45] Naik NG, Wu HN (2015). Mutation of Putative N-Glycosylation Sites on Dengue Virus NS4B Decreases RNA Replication. J Virol.

[46] Neupane B (2021). Murine Trophoblast Stem Cells and Their Differentiated Cells Attenuate Zika Virus In Vitro by Reducing Glycosylation of the Viral Envelope Protein. Cells.

[47] Reszka N (2010). Effect of tunicamycin on the biogenesis of hepatitis C virus glycoproteins. Acta Biochim Pol.

[48] Dawood AA, Altobje MA (2020). Inhibition of N-linked Glycosylation by Tunicamycin May Contribute to The Treatment of SARS-CoV-2. Microb Pathog.

[49] Santos-Beneit F (2021). A metabolic modeling approach reveals promising therapeutic targets and antiviral drugs to combat COVID-19. Sci Rep.

[50] Shan C (2016). An Infectious cDNA Clone of Zika Virus to Study Viral Virulence, Mosquito Transmission, and Antiviral Inhibitors. Cell Host Microbe.

