# Supplementary material for: The glycosylation deficiency of flavivirus NS1 attenuates virus replication through interfering with the formation of viral replication compartments
Source: J Biomed Sci. 2024 Jun 7;31:60. doi: 10.1186/s12929-024-01048-z (PMC11157723; doi:10.1186/s12929-024-01048-z)
Supplement: Supplementary file 1 — Supplementary Material 1. [file 12929_2024_1048_MOESM1_ESM.docx]

**
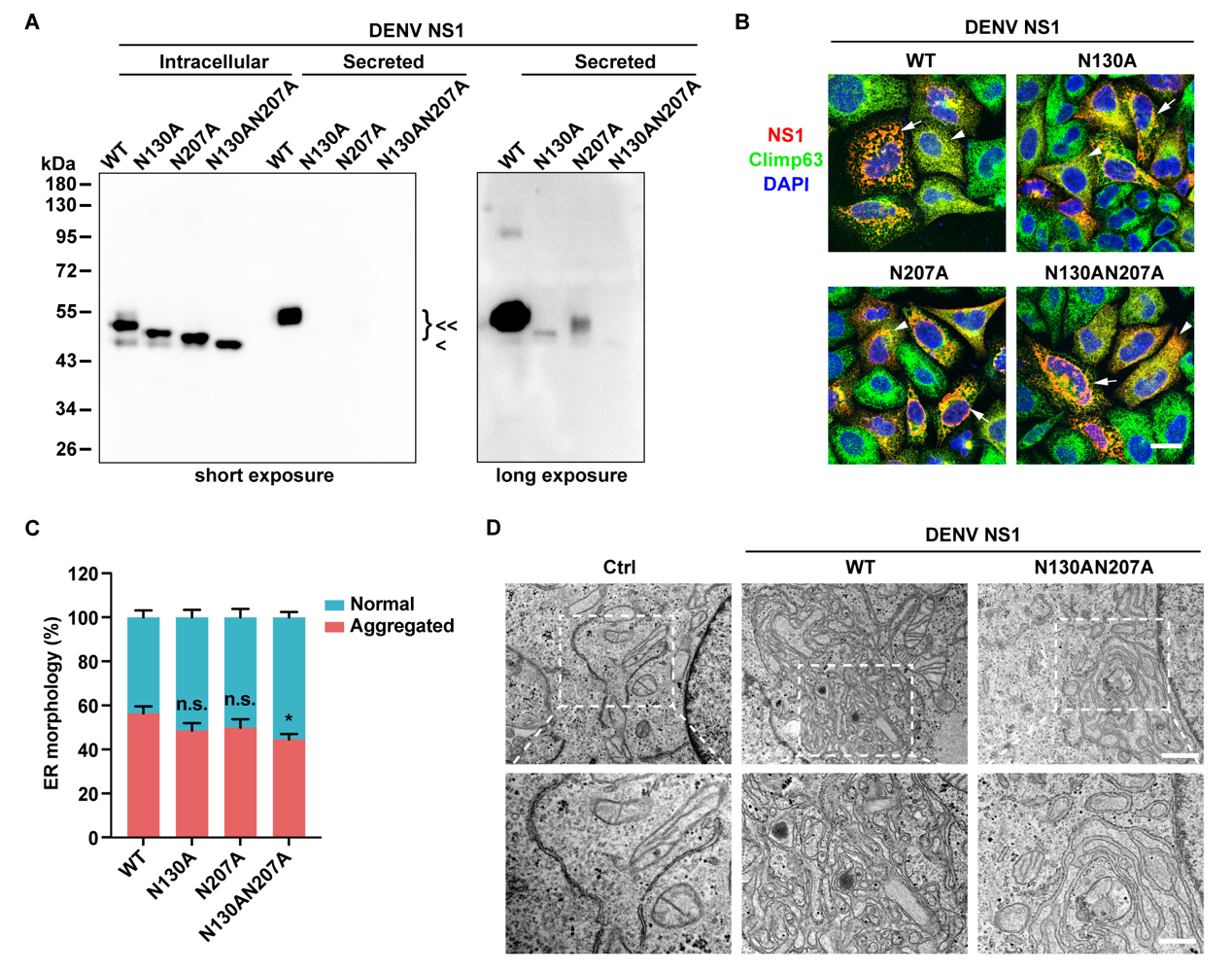
**

**Fig. S1. N-linked glycosylation of DENV NS1 is important for ER remodeling**

**(A)** The glycosylation levels of WT and mutated DENV NS1. Intracellular and secreted DENV NS1 expressed in HeLa cells was analyzed by Western blotting. <<, glycosylated NS1; <, non-glycosylated NS1 (N130AN207A). **(B)** NS1 induced ER morphology changes. HeLa cells expressing Myc-tagged WT or mutated DENV NS1 were stained with Myc and climp63 antibodies, with climp63 serving as the ER marker. NS1-positive cells were categorized into two groups based on ER morphology. “Aggregated ER” denotes ER aggregations above 5 μm^2^, as indicated by arrows; while “normal ER” indicates the ER maintaining sheet and tubule structures with ER aggregations below 5 μm^2^, as indicated by arrowheads. Scale bar, 20 μm. **(C)** Quantification of cell population with different ER morphologies. The percentage was calculated as the number of cells with aggregated ER divided by the total number of NS1-positive cells. Values are the means ± SEM of three technical replicates (*, p<0.05; n.s., non-significant; two-tailed t test). **(D)** Ultrastructure of ER in HeLa cells overexpressing WT and mutated DENV NS1 was captured by TEM. Ctrl, untransfected cells. Scale bar, 1 μm (upper), 500 nm (lower).


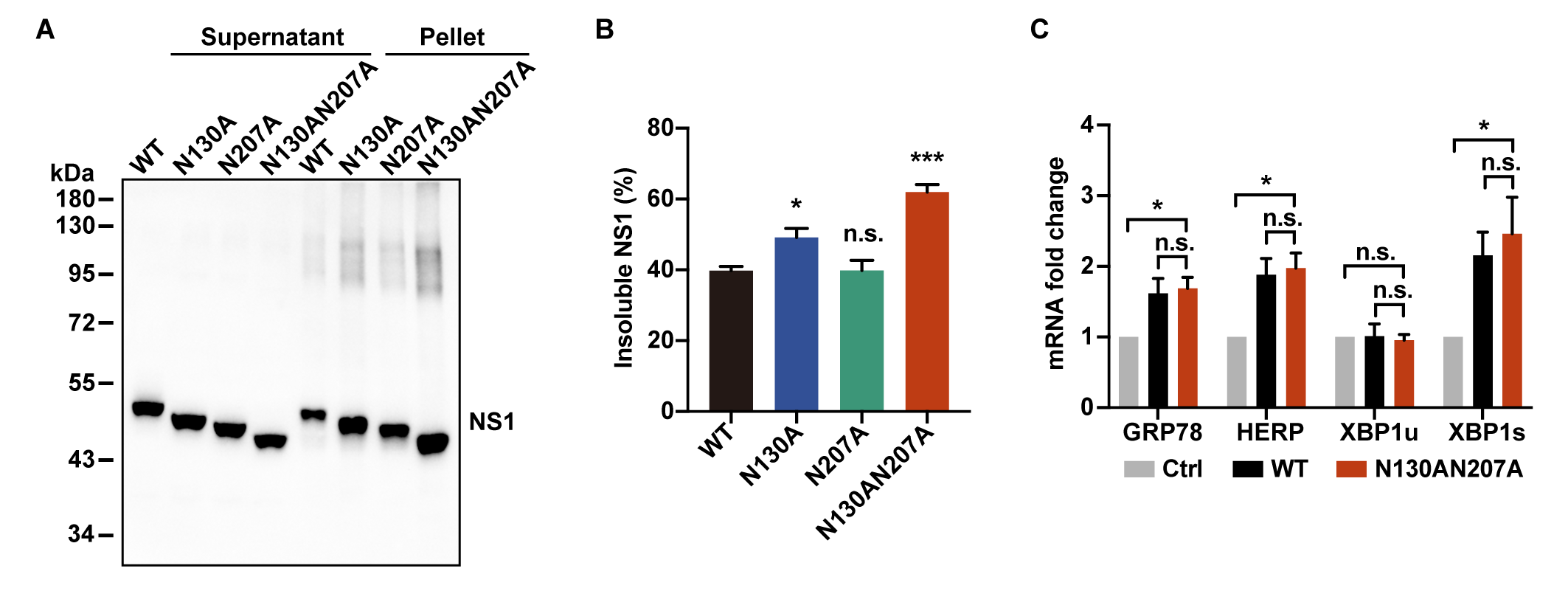


**Fig. S2. Glycosylation deficiency of DENV NS1 leads to protein aggregation**

**(A)** Glycosylation deficiency led to DENV NS1 aggregation. HeLa cells were transfected with plasmids encoding for WT or mutated DENV NS1 proteins, and the soluble lysates and detergent-resistant pellets were analyzed by Western blotting. **(B)** Quantification of insoluble NS1 (detergent-resistant pellet) in (A). The percentage was calculated as insoluble NS1 divided by the total NS1 (the sum of soluble and insoluble NS1). **(C)** DENV NS1 induced ER stress. Total RNA was extracted from HeLa cells and the mRNA levels of ER stress sensors were examined by qPCR. Ctrl, untransfected cells. All statistical data represent mean ± SEM (n=3; *, p<0.05; ***, p<0.001; n.s., non-significant; two-tailed t test)


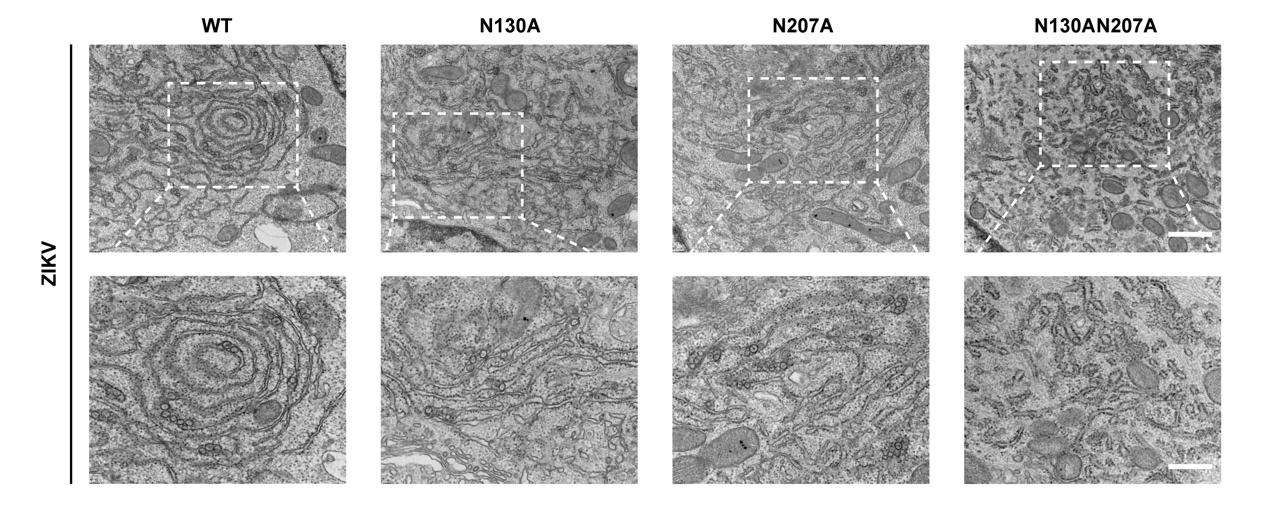


**Fig. S3. Glycosylation deficiency of ZIKV NS1 inhibited RCs formation in ZIKV-infected HeLa cells**

Ultrastructure of the ER in HeLa cells infected with WT or mutated ZIKV was captured by TEM. Scale bar, 1 μm (upper), 500 nm (lower).


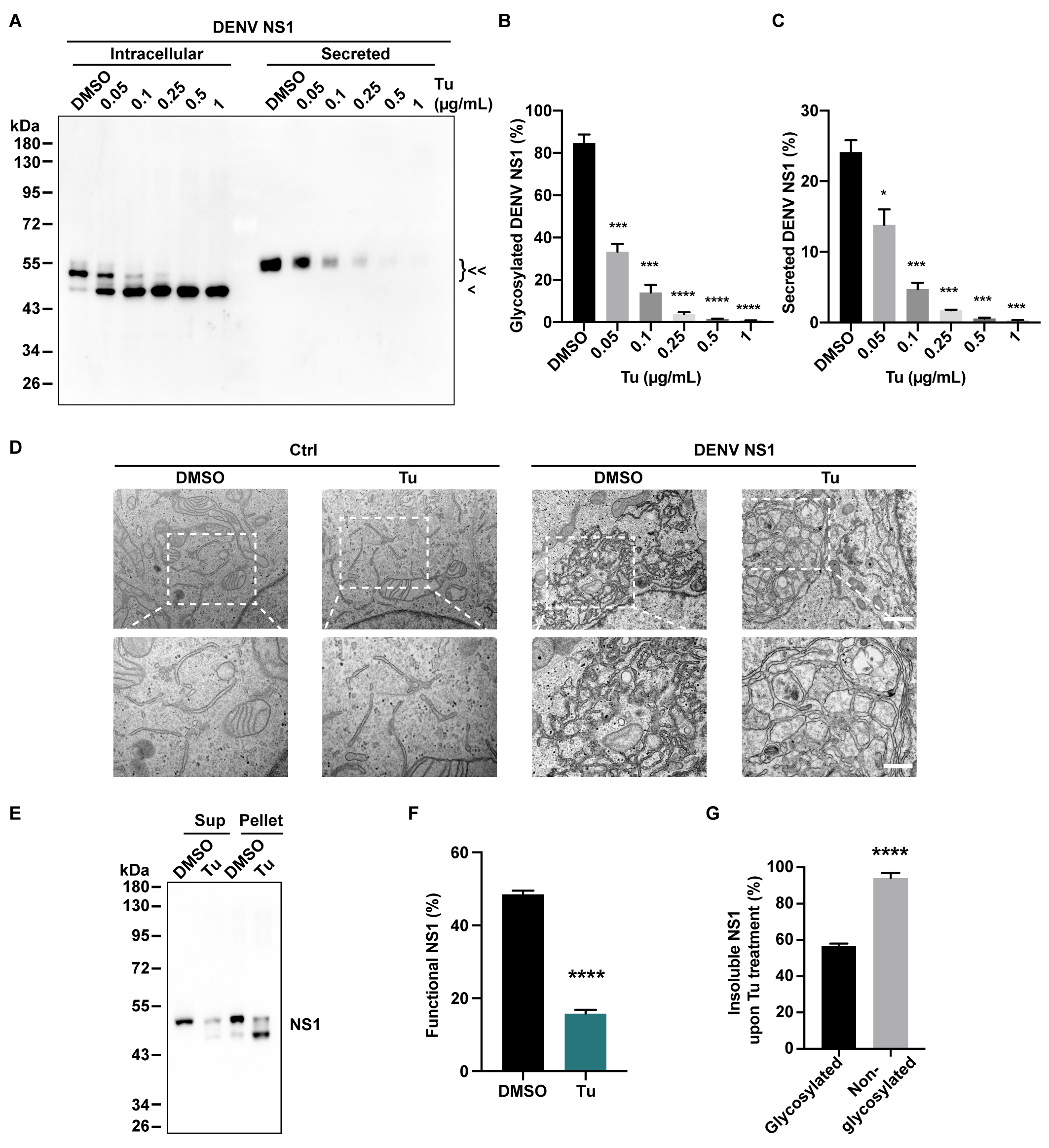


**Fig. S4. Tunicamycin blocks DENV NS1 glycosylation and interferes with its ER remodeling**

(**A**) The glycosylation levels of DENV NS1 under Tu treatment. Intracellular and secreted NS1 proteins expressed in HeLa cells were immunoblotted with a Myc antibody. Intracellular samples were diluted 5 times with protein loading buffer. <<, glycosylated NS1; <, non-glycosylated NS1. DMSO as a control. (**B**) Quantification of glycosylated NS1. The percentage was calculated as glycosylated NS1 divided by intracellular NS1. (**C**) Quantification of secreted NS1. The percentage was calculated as secreted NS1 divided by the total NS1 (the sum of intracellular and secreted NS1). **(D)** TEM images showed the ER ultrastructure in HeLa cells transfected with DENV NS1 WT plasmid under 0.25 μg/mL Tu treatment. Scale bar, 1 μm (upper), 500 nm (lower). **(E)** Tu induced DENV NS1 aggregation. HeLa cells were transfected with WT DENV NS1 plasmid and treated with 0.5 μg/mL Tu, and the soluble lysates and detergent-resistant pellets were analyzed by Western blotting. **(F)** Quantification of soluble functional NS1 (detergent-soluble supernatant) in (E). The percentage was calculated as soluble NS1 in the supernatant divided by the total NS1 (the sum of soluble and insoluble NS1). **(G)** Quantification of glycosylated or non-glycosylated insoluble NS1 upon Tu treatment in (E). The percentage of insoluble NS1 was calculated by dividing glycosylated or non-glycosylated insoluble NS1 by the total glycosylated or non-glycosylated NS1, respectively. All statistical data represent mean ± SEM (n=3; *, p<0.05; ***, p<0.001; ****, p<0.0001; two-tailed t test).


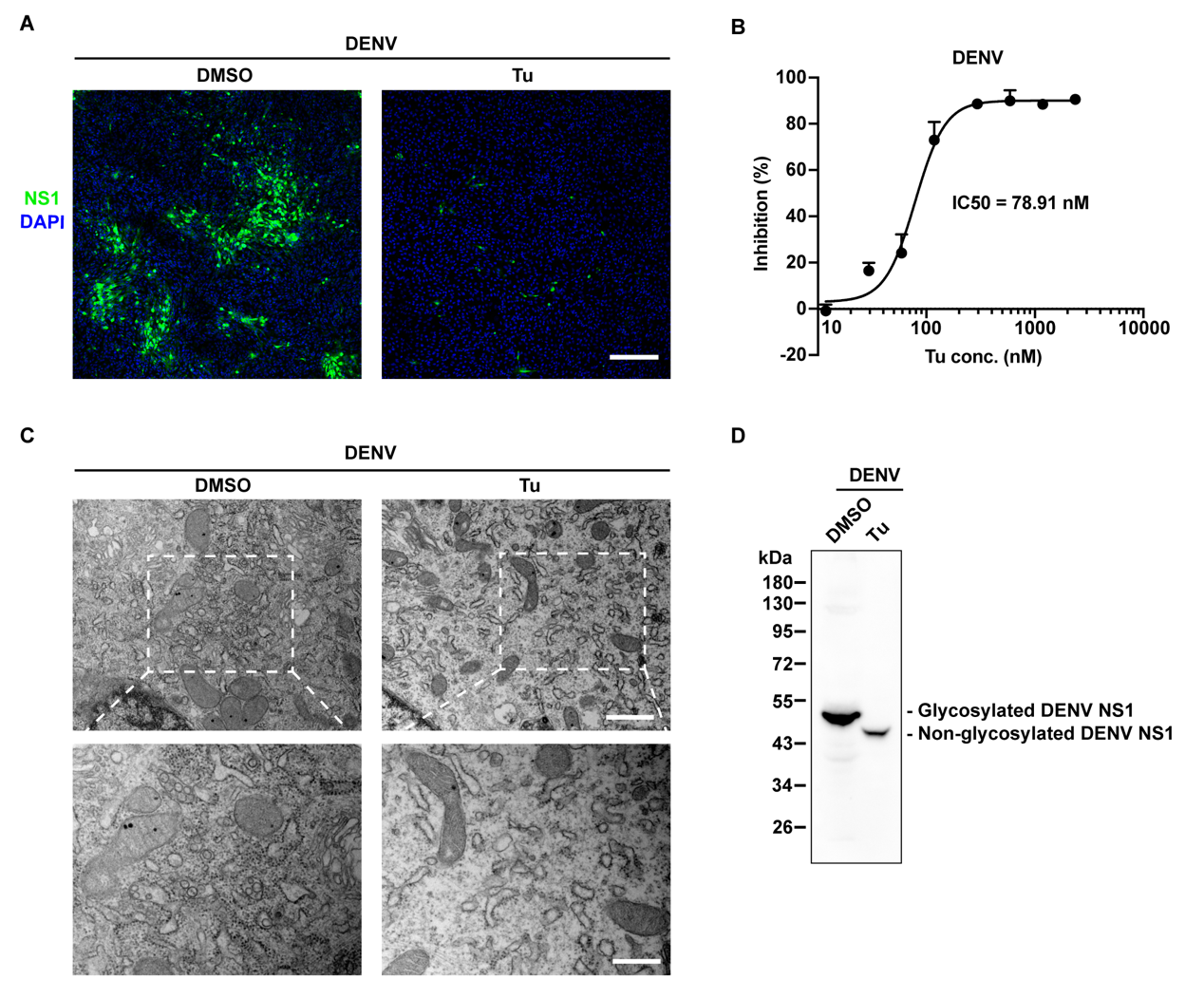


**Fig. S5. Tunicamycin inhibits DENV replication**

(**A**) Tu inhibited DENV replication. DENV-infected BHK-21 cells were treated with Tu (0.25 μg/mL) at 8 hpi and stained with an NS1 antibody at 24 hpi. Scale bar, 100 μm. (**B**) IC50 curve of Tu for DENV replication. DENV replication levels in BHK-21 cells under Tu treatment were quantified by qPCR at 24 hpi. The IC50 value is 78.91 nM. Values are the mean ± SEM. (**C**) Tu inhibited DENV RCs formation. DENV RCs in BHK-21 cells were captured by TEM. Scale bar, 1 μm (upper), 500 nm (lower). (**D**) The glycosylation status of DENV NS1 in DENV-infected BHK-21 cells treated with Tu was detected by western blotting with NS1 antibody at 24 hpi.
