# Supplementary material for: The glycosylation deficiency of flavivirus NS1 attenuates virus replication through interfering with the formation of viral replication compartments
Source: J Biomed Sci. 2024 Jun 7;31:60. doi: 10.1186/s12929-024-01048-z (PMC11157723; doi:10.1186/s12929-024-01048-z)
Supplement: Supplementary file 3 — Supplementary Material 3. [file 12929_2024_1048_MOESM3_ESM.pdf]

SPRINGER NATURE

Change of authorship request form - Journals (pre-acceptance)

Important information. Please read.

- This form should be used by authors to request any change in authorship (adding/deleting authors) including changes in corresponding authors. This form should not be used for name changes. Please fully complete all sections. Use black ink and block capitals and provide each author's full name with the given name first followed by the family name.

- By signing this declaration, all authors guarantee that the order of the authors are in accordance with their scientific contribution, if applicable as different conventions apply per discipline, and that only authors have been added who made a meaningful contribution to the work.

- Please note, in author collaborations where there is formal agreement for representing the collaboration, it is sufficient for the representative or legal guarantor (usually the corresponding author) to complete and sign the Authorship Change Form on behalf of all authors, **next to the added/removed author(s). (Complete Section 3, followed by Section 6.)**
  In author collaborations where there is no formal agreement for representing the collaboration and **there are more than 10 authors**, one may sign for all, provided the signer appends correspondence that attests that each of the authors have agreed to the change **and the added/removed authors sign the form. (Complete Section 3, followed by Section 6.)**

- Please note, we cannot investigate or mediate any authorship disputes. If you are unable to obtain agreement from all authors (including those who you wish to be removed) you must refer the matter to your institution(s) for investigation. Please inform us if you need to do this.

- If you are not able to return a fully completed form within **30 days** of the date that it was sent to the author requesting the change, we may have to withdraw your manuscript. We cannot publish manuscripts where authorship has not been agreed by all authors (including those who have been removed).

- Incomplete forms will be rejected.

- Please return/upload this form, fully completed, to the Journals Editorial Office. The Journal and/or Publisher will consider the information you have provided to decide whether to approve the proposed change in authorship. We may decide to contact your institution for more information or undertake a further investigation, if appropriate, before making a final decision.

SPRINGER NATURE **Change of authorship request form - Journals** (pre-acceptance)

Section 1: Please provide the current title of manuscript

Manuscript ID no.: JBMS-D-23-03066

Title: The glycosylation deficiency of flavivirus NS1 attenuates virus replication through interfering with the formation of viral replication compartments

Section 2: Please provide the previous authorship, in the order shown on the manuscript before the changes were introduced. Please indicate the corresponding author by adding (CA) behind the name.

	First name(s)	Family name	ORCID or SCOPUS id, if available
1st author	Yali	Ci (CA)	
2nd author	Shuhan	Huang	
3rd author	Pan-Deng	Shi	
4th author	Xiao-Xuan	Fan	
5th author	Yang	Yang	
6th author	Cheng-Feng	Qin	
7th author	Hui	Zhao (CA)	
8th author	Lei	Shi (CA)	
9th author			
10th author			

Please use an additional sheet if there are more than 10 authors.

SPRINGER NATURE Springer Nature is one of the world's leading global research, educational and professional publishers, created in May 2015 through the combination of Nature Publishing Group, Palgrave Macmillan, Macmillan Education and Springer Science+Business Media.

SPRINGER NATURE

Change of authorship request form - Journals (pre-acceptance)

Section 3: Please provide a justification for change. Please use this section to explain your reasons for changing the authorship of your manuscript, e.g. what necessitated the change in authorship? Please refer to the (journal) policy pages for more information about authorship. Please explain why omitted authors were not originally included and/or why authors were removed on the submitted manuscript.

We need to rearrange the order of authorship without adding or removing any authors, reflecting their contributions during the revision process.

Section 4: Proposed new authorship. Please provide your new authorship list in the order you would like it to appear on the manuscript. Please indicate the corresponding author by adding (CA) behind the name. If the Corresponding Author has changed, please indicate the reason under section 3.

	First name(s)	Family name (this name will appear in full on the final publication and will be searchable in various abstract and indexing databases)	Affiliated institute	E-mail address
1st author	Shuhan	Huang	Institute of Basic Medical Sciences, Chinese Academy of Medical Sciences and School of Basic Medicine	hshhyn19971002@163.com
2nd author	Pan-Deng	Shi	Beijing Institute of Microbiology and Epidemiology, Academy of Military Medical Sciences	shi_pd@126.com
3rd author	Xiao-Xuan	Fan	Beijing Institute of Microbiology and Epidemiology, Academy of Military Medical Sciences	fanxx1999@163.com
4th author	Yang	Yang	Institute of Basic Medical Sciences, Chinese Academy of Medical Sciences and School of Basic Medicine	yangy@ibms.pumc.edu.cn
5th author	Cheng-Feng	Qin	Beijing Institute of Microbiology and Epidemiology, Academy of Military Medical Sciences	qincf@bmi.ac.cn
6th author	Hui	Zhao (CA)	Beijing Institute of Microbiology and Epidemiology, Academy of Military Medical Sciences	shuishu2002@126.com
7th author	Lei	Shi (CA)	Institute of Basic Medical Sciences, Chinese Academy of Medical Sciences and School of Basic Medicine	shilei@ibms.pumc.edu.cn
8th author	Yali	Ci (CA)	Institute of Basic Medical Sciences, Chinese Academy of Medical Sciences and School of Basic Medicine	ciyali@ibms.pumc.edu.cn
9th author				
10th author				

Please use an additional sheet if there are more than 10 authors.

SPRINGER NATURE Springer Nature is one of the world's leading global research, educational and professional publishers, created in May 2015 through the combination of Nature Publishing Group, Palgrave Macmillan, Macmillan Education and Springer Science+Business Media.

SPRINGER NATURE Change of authorship request form - Journals (pre-acceptance)

Section 5: Author contribution, Acknowledgement and Disclosures. Please use this section to provide a new disclosure statement and, if appropriate, acknowledge any contributors who have been removed as authors and ensure you state what contribution any new authors made (if applicable per the journal or book (series) policy). Please ensure these are updated in your manuscript - after approval of the change(s) - as our production department will not transfer the information in this form to your manuscript.

New acknowledgements:

We would like to thank Dr. Peiyong Shi (University of Texas Medical Branch, USA) for kindly providing ZIKV infectious DNA clone and Dr. Bo Zhang (Institute of Virology, Chinese Academy of Sciences, Wuhan, China) for providing ZIKV replicon clone. We are grateful to the Cell Biology Facility affiliated with the Center of Biomedical Analysis, Tsinghua University, for technical assistance and equipment support.

New Disclosures (financial and non-financial interests, funding):

Financial and non-financial interests:

The authors declare no competing interests.

Funding:

We thank the funding from, National Natural Science Foundation of China (82272328 and 81622028 to LS, 32200551 to YC), CAMS Innovation Fund for Medical Sciences (CIFMS) 2022-I2M-1-011.

New Author Contributions statement (if applicable per the journal policy):

YC, HZ and LS conceived the project. SH, YC, PS, XF and HZ performed experiments. YC and SH contributed in experiments and data analysis. YC and LS wrote the manuscript and all other authors edited the manuscript.

State 'Not applicable' if there are no new authors.

SPRINGER NATURE Springer Nature is one of the world's leading global research, educational and professional publishers, created in May 2015 through the combination of Nature Publishing Group, Palgrave Macmillan, Macmillan Education and Springer Science+Business Media.

SPRINGER NATURE Change of authorship request form - Journals (pre-acceptance)

Section 6: Declaration of agreement. *All* authors, unchanged, new and removed *must* sign this declaration.
(NB: Please print the form, (docu)-sign and return/upload a scanned copy. Please note that signatures that have been inserted as an image file are acceptable as long as it is handwritten. Typed names in the signature box are unacceptable.) *** Please delete as appropriate. Delete all of the bold if you were on the original authorship list and are remaining as an author.**

	First name	Family name		Signature	Date
1st author	SHUHAN	HUANG	I agree to the proposed new authorship shown in section 4 /~~**and the addition/removal*of my name to the authorship list**~~/and the proposed change in corresponding author	Shuhan Huang	2024.05.17
2nd author	PAN-DENG	SHI	I agree to the proposed new authorship shown in section 4 /~~**and the addition/removal*of my name to the authorship list**~~/and the proposed change in corresponding author	Pandeng Shi	2024,05.17
3rd author	XIAO-XUAN	FAN	I agree to the proposed new authorship shown in section 4 /~~**and the addition/removal*of my name to the authorship list**~~/and the proposed change in corresponding author	Xiao xuan Fan	2024.05.17
4th authors	YANG	YANG	I agree to the proposed new authorship shown in section 4 /~~**and the addition/removal*of my name to the authorship list**~~ /and the proposed change in corresponding author	Yangyang	2024.5.17
5th author	CHENG-FENG	QIN	I agree to the proposed new authorship shown in section 4 /~~**and the addition/removal*of my name to the authorship list**~~/and the proposed change in corresponding author	Chengfeng Qin	2024.5.17
6th author	HUI	ZHAO	I agree to the proposed new authorship shown in section 4 /~~**and the addition/removal*of my name to the authorship list**~~/and the proposed change in corresponding author	Hui Zhao	2024.5.17
7th author	LEI	SHI	I agree to the proposed new authorship shown in section 4 /~~**and the addition/removal*of my name to the authorship list**~~/and the proposed change in corresponding author	Lei Shi	2024.5.17

SPRINGER NATURE Springer Nature is one of the world's leading global research, educational and professional publishers, created in May 2015 through the combination of Nature Publishing Group, Palgrave Macmillan, Macmillan Education and Springer Science+Business Media.

SPRINGER NATURE 	# Change of authorship request form - Journals (pre-acceptance)

	First name	Family name		Signature	Date
8th author	YALI	CI	I agree to the proposed new authorship shown in section 4 /~~and the addition/removal*of my name to the authorship list~~ /and the proposed change in corresponding author	Yali Ci	2024.5.17
9th author			I agree to the proposed new authorship shown in section 4 /**and the addition/removal*of my name to the authorship list** /and the proposed change in corresponding author		
10th author			I agree to the proposed new authorship shown in section 4 /**and the addition/removal*of my name to the authorship list** /and the proposed change in corresponding author		

Please use an additional sheet if there are more than 10 authors.

In case of author collaborations with formal agreement:

.	Name of consortium/consortia	First name	Family name		Signature	Date
Representative/legal guarantor				I agree to the proposed new authorship shown in section 4 /**and the addition/removal*of my name to the authorship list** /and the proposed change in corresponding author		

Both added/removed authors should complete the information in the first table under Section 6.

---- End of form ----

SPRINGER NATURE Springer Nature is one of the world's leading global research, educational and professional publishers, created in May 2015 through the combination of Nature Publishing Group, Palgrave Macmillan, Macmillan Education and Springer Science+Business Media.
