# Supplementary material for: The glycosylation deficiency of flavivirus NS1 attenuates virus replication through interfering with the formation of viral replication compartments
Source: J Biomed Sci. 2024 Jun 7;31:60. doi: 10.1186/s12929-024-01048-z (PMC11157723; doi:10.1186/s12929-024-01048-z)
Supplement: Supplementary file 2 — Supplementary Material 2. [file 12929_2024_1048_MOESM2_ESM.docx]

Dr. Ben Berkhout

Editor-in-chief

Journal of Biomedical Science

Dear Dr. Berkhout,

Re: Resubmission of manuscript reference No. JBMS-D-23-03066.

Here we submit a revised version of our manuscript entitled “The glycosylation deficiency of flavivirus NS1 attenuates virus replication through interfering with the formation of viral replication compartments”.

We sincerely thank the editor and all reviewers for their valuable feedback that we have used to improve our manuscript. A point-to-point response to reviewer comments is attached with revised manuscript. We hope that our revisions would strengthen our work sufficiently and fully address reviewers’ questions. We marked the changes with “RED” color in our revised manuscript.

We hope the revised version is now suitable for publication and look forward to your positive response.

Sincerely,

Yali Ci & Lei Shi,

CAMS & PUMC

**Point-to-Point Response**

Reviewer # 1:

1. The data presented with the infectious clones is very limited. Authors should try to reproduce with the infectious clones some of the results obtained with the recombinant ZIKV NS1 proteins. For example, are the changes observed by TEM and presented in Figures 1E and 5F also observed with the mutated infectious clones? Are there other morphological changes observed in the ER of the cells transfected with the infectious clones? Addition of these observations will greatly strengthen this manuscript.

Thank you for your suggestion. We observed and analyzed the ER morphologies in both BHK-21 cells (Fig. 4F in the revised manuscript) and HeLa cells (Fig. S3 in the revised supplemental material) infected with wide-type recombinant ZIKV (rZIKV) or mutated rZIKV by TEM. The results indicated that N130A and N207A mutations can still induce RCs but decrease the number of RCs, while the double point mutation N130AN207A resulted in a significant inhibition of RCs formation in both BHK-21 and HeLa cells (Figure 1), supporting that NS1 glycosylated mutations inhibit viral replication (Fig. 4A-E in the revised manuscript).

**Figure 1. Glycosylation deficiency of NS1 inhibits RCs formation in rZIKV-infected cells**

The ultrastructure of the ER in BHK-21 cells **(A)** and HeLa cells **(B)** infected with WT or mutated rZIKV was captured by TEM. Scale bar, 1 μm (upper), 500 nm (lower).

2. Authors should briefly describe the morphological criteria used to define an "aggregated ER" versus a "normal" ER in the studied cells; this to better define what is meant by "remodeling capacity" of the NS1 over the ER structure.

Thank you for the suggestion. We have described the morphological criteria in the legend of Fig. 1C in the manuscript: “NS1-positive cells were categorized into two groups based on ER morphology. ‘Aggregated ER’ denotes ER aggregations above 5 μm^2^, as indicated by arrows, while ‘normal ER’ indicates the ER maintaining sheet and tubule structures with aggregations below 5 μm^2^, as indicated by arrowheads.” (p.24, lines 671-673 in the manuscript). We utilized ImageJ software to measure the size of the ER aggregations.

3. In the cells treated with Tunicamycin, did the authors evaluate the glycosylation state of E protein? Can the effect of Tunicamycin on the E protein contribute to the phenotype they are all attributing to NS1?

Thanks for the question. Accordingly, we examined the glycosylation state of the E protein in cells treated with Tunicamycin (Tu) using Western blotting. We observed a band corresponding to E protein, slightly lower in molecular weight compared to untreated cells, indicating a deficiency in glycosylation (Figure 2A and 2B). Given that the E protein possesses only one glycosylation site, the observed change in apparent molecular weight of E is smaller than that of NS1 (Figure 2B)[1]. Importantly, we found no significant alterations in ER morphology upon overexpression of E, regardless of its glycosylation status (Figure 2C). These findings suggest that the ER remodeling induced by ZIKV infection is independent of the E protein, and that the deficiency in glycosylation of E does not affect ER rearrangement either. It is also widely recognized that the E protein is mostly involved in virus entry and fusion steps, not directly in virus replication. Our results strongly suggest that NS1 plays a pivotal role in ER remodeling process, although we cannot discount the involvement of other NS proteins in the formation of ZIKV replication compartments (RCs).

**Figure 2. E protein does not affect the ER morphology**

**(A and B)** Tu inhibits the glycosylation of the E protein. HeLa cells were either transfected with the E plasmid **(A)** or infected with ZIKV **(B)**, then treated with DMSO or 0.25 μg/mL Tu. Glycosylation of the E protein and NS1 protein was analyzed by Western blotting with E and NS1 antibodies respectively. **(C)** E protein does not affect the ER morphology with/without Tu treatment. HeLa cells transfected with the E plasmid and treated with DMSO or 0.25 μg/mL Tu were immunostained with E and climp63 antibodies. Scale bar: 20 μm.

4. Figure 4B. The attenuation in virus RNA replication claimed by the authors is not evident. Authors should find a more representative image or even better present the result of a quantification of several images.

Thanks for your kind suggestion. To further support our argument, we quantified the number of dsRNA-positive cells across 30 fields from three independent experiments. The results are presented in Fig. 4C in the manuscript. Our quantitative analysis indicated that glycosylation mutations of the NS1 protein reduced the dsRNA-positive rate, with the double mutation (N130AN207A) showing the most pronounced inhibitory effect (Figure 3).

**Figure 3. NS1 glycosylation mutations on ZIKV replicons inhibit viral replication**

**(A)** Immunofluorescence assay detecting replication efficiency. BHK-21 cells transfected with ZIKV replicons were stained with a dsRNA antibody (Green: dsRNA, Blue: DAPI). Scale bar, 20 μm. **(B)** Quantification of dsRNA-positive cells. Statistical analysis was performed from three independent experiments by analyzing 30 fields for each group. WT ZIKV replicon is set as 100%. All statistical data represent mean ± SEM (n=3; ***, p<0.001; ****, p<0.0001; two-tailed t test).

5. Figures 1A and B. How was the protein load for these WB controlled for? Please explain.

Thanks for this suggestion. We have added protein quantification in the Materials and Methods section (p.7, lines 162-181 in the manuscript). For Fig. 1A and 1B in the manuscript, an equal number of cells were initially seeded in a 24-well plate, followed by the transfection of equal amounts of plasmids into each well. At 8 hours post-transfection (hpt), the cell culture medium was replaced with 300 μL Opti-MEM. At 24 hpt, 300 μL of supernatant was harvested as the secreted sample, and the cells were lysed with 100 μL of 1×SDS-PAGE loading buffer. For Western blotting, 6 μL of cell lysate and 18 μL of supernatant were loaded onto the gel. We calculated the percentage of secreted NS1 protein by dividing the amount of secreted NS1 by the sum of intracellular NS1 and secreted NS1 proteins.

6. Authors may be interested in the following work; Choy et al. Cell Reports. 2020. 31 (6): 107617.

Thank you for this helpful suggestion. Choy et al. revealed that G53D mutation on DENV NS1 disrupted NS1 protein glycosylation, resulting in reduced virus infection and dissemination. Their findings highlighted the significance of NS1 glycosylation on flavivirus infection, which aligns with our conclusion. We have cited the paper in the discussion section (seen as manuscript Ref. 38).

Reviewer # 2:

1. The link between NS1 glycosylation and virus replication complex formation is not supported by the current data. The modest effects of NS1 N-linked glycosylation mutants on overall virus replication (only a 2-fold reduction with the double mutant - Figure 4a) suggests that in the absence of glycosylation, there is still a significant level of virus replication. Therefore, NS1 glycosylation does not appear to be strictly necessary for virus replication complex formation, which is a requirement for virus replication. Rather, the data indicate that the effect of NS1 mutants on membrane reorganization is indirect, caused either by the increased NS1 in insoluble fractions or by the increased ER stress. Mutants in the NS1 glycosylation sites cause accumulation in insoluble ER fractions but the portion of NS1 that is not in the insoluble fraction is still able to mediate virus replication without N-linked glycosylation.

Thank you for your comment. In our manuscript, “RCs” means replication compartments, distinct from viral replication complexes. Viral replication complexes (located in the cytosolic side of the ER) comprise NS3-NS5 and other NS proteins for viral RNA synthesis. NS1 (located in the ER lumen) participates in the formation of replication compartments, which are membranous vesicular structures encapsulating viral replication complexes. We believe we provide strong evidence demonstrating that NS1 glycosylation is necessary and significant for the formation of viral replication compartments, thereby impacting viral replication.

In our earlier study, we revealed the essential role of NS1 in the formation of viral replication compartments and identified more severe NS1 mutations that nearly completely block virus replication[2], establishing a connection between NS1 and virus replication. Here, in both ZIKV replicon assay and plaque assay, NS1 glycosylation mutations showed inhibitory effects, with the double mutation (N130AN207A) being the most severe (Fig. 4A-E in the revised manuscript). Yes, we agree that glycosylation mutations of NS1 are not lethal (or highly significant), but they do **attenuate** virus replication rather than completely block it. Moreover, our results are consistent with earlier reports from Dr. [Michael Diamond](https://pubmed.ncbi.nlm.nih.gov/?sort=date&size=100&term=Diamond+MS&cauthor_id=28708997) lab, Dr. [Charles](https://pubmed.ncbi.nlm.nih.gov/?sort=date&size=100&term=Diamond+MS&cauthor_id=28708997) Rice lab, and others[3-5], although those studies provided limited mechanistic insights. For example, Figure 4 corresponds to “Figure 2C-E” cited from the paper “Vaccine Mediated Protection Against Zika Virus-Induced Congenital Disease” (Cell. 2017 Jul 13;170(2):273-283 by [Michael S Diamond](https://pubmed.ncbi.nlm.nih.gov/?sort=date&size=100&term=Diamond+MS&cauthor_id=28708997) lab), which also demonstrated similar inhibitory effects (not that highly significant) on ZIKV replication by NS1 glycosylation site mutations. Building upon this, they developed and tested a live-attenuated ZIKV vaccine with N-glycosylation site mutations on NS1. We think that evidence from both our current study and other groups supports the notion that NS1 glycosylation is required for viral replication.

**Figure 4.** Attenuated growth of live-attenuated ZIKV vaccine with NS1-DKO mutations

Plaque assays **(C)**, replication kinetics **(D)**, and transient replicon **(E)** assays were performed in Vero cells. This figure is cited from Figure 2 C-E in “Vaccine Mediated Protection Against Zika Virus-Induced Congenital Disease” (Cell. 2017 Jul 13;170(2):273-283).

Glycosylation deficiency of NS1 do not directly impact its membrane binding and remodeling abilities. Instead, it interferes NS1 stability, resulting in reduced functional NS1 levels and the accumulation of non-glycosylated NS1 in the pellet fraction, ultimately impairing viral replication (Figure 5). We think that we provide strong evidence showing that glycosylation of NS1 affects the formation of viral replication compartments. The limited induction of ER stress by WT or mutated NS1 may contribute to the attenuation of viral replication, but it likely plays a subsidiary role.

**Figure 5. Tunicamycin reduces functional NS1 and non-glycosylated NS1 tends to accumulate in the pellet**

**(A)** HeLa cells were transfected with WT ZIKV NS1 plasmid and treated with 0.5 μg/mL Tu. Then the soluble supernatants and detergent-resistant pellets were analyzed by Western blotting. **(B)** Quantification of functional NS1 (detergent-soluble supernatant) in (A). The percentage was calculated as soluble NS1 in the supernatant divided by the total NS1 (the sum of soluble and insoluble NS1). **(C)** Quantification of glycosylated or non-glycosylated insoluble NS1 upon Tu treatment in (A). The percentage of insoluble NS1 was calculated by dividing glycosylated or non-glycosylated insoluble NS1 by the total glycosylated or non-glycosylated NS1, respectively. **(D)** HeLa cells were transfected with WT DENV NS1 plasmid and treated with 0.5 μg/mL Tu, and the soluble supernatants and detergent-resistant pellets were analyzed by Western blotting. **(E)** Quantification of functional NS1 in (D). **(F)** Quantification of glycosylated or non-glycosylated insoluble NS1 upon Tu treatment in (D). All statistical data represent mean ± SEM (n=3; ***, p<0.001; ****, p<0.0001; two-tailed t test).

We have demonstrated that a higher proportion of non-glycosylated NS1 accumulated in the insoluble fraction, indicating the instability of non-glycosylated NS1 and the decrease of functional NS1 (Figure 5). While we agree that “the portion of NS1 that is not in the insoluble fraction is still able to mediate virus replication without N-linked glycosylation”, due to the underdose of functional NS1, the formation of viral RCs was inhibited, resulting in the attenuation of virus replication. Thus, glycosylation of NS1 is required and important for efficient virus replication.

2. Further to my first point, in Figure 1 the authors show that all NS1 N-linked glycosylation mutants lead to significant changes in ER morphology compared to wildtype. However, there is little effect of N130A or N270A on virus replication or titers in Figure 4. This indicates that the effects seen on ER morphology are not directly linked to virus replication.

Thank you for the comment. Single mutations of NS1 glycosylation sites (N130A and N207A) exhibited a relatively mild phenotype in altering ER morphology under TEM (Fig. 1E and 1F in the revised manuscript), as well as in virus replication, both in our study and in the report by Michael S. Diamond group (see Figure 4C). However, the double mutation of NS1 glycosylation sites (N130AN207A) showed a more significant effect on changing ER morphology, which also displayed stronger inhibition of virus replication. In our earlier study, we had shown that flavivirus NS1 could remodel the host ER and play a critical role in the formation of flavivirus replication compartments, thus affecting virus replication[2]. Considering these results collectively, we believe that NS1 mediated ER remodeling is crucial for flavivirus replication. The glycosylation affects NS1 stability and viral replication compartments formation, thereby inhibiting virus replication. We think our data successfully established the linkage between NS1 glycosylation, ER remodeling and virus replication.

3. Tunicamycin is commonly used to induce a UPR response in cells and has previously been shown to limit Zika virus replication (See PMID: 34106769, 34831310). The effect on Zika virus replication has been linked to both UPR activation and envelop protein glycosylation. Although the current data show that NS1 is not glycosylated in Tunicamycin-treated cells, it is not clear from the current data if the effect on virus replication is linked to NS1 glycosylation. The authors demonstrate how their analysis adds to this body of literature.

Thanks for the comment. The first paper (PMID: 34106769) suggests that tunicamycin (Tu) can induce the UPR response, and the second paper suggests that Tu blocks the glycosylation of the ZIKV E protein, thereby inhibiting viral replication. Our another study suggested that the UPR response and ERAD pathways play an important role in flavivirus replication[6]. However, this does not imply that Tu inhibits flavivirus replication solely through the UPR pathway. We agree that Tu inhibits flavivirus replication via multiple mechanisms, including affecting protein glycosylation and the UPR response activation. Regarding the E protein, as showed in Figure 2, we also observed hypo-glycosylation of the E protein under Tu treatment, and the E protein did not change ER morphology with or without Tu treatment. It is also widely recognized that the E protein is mostly involved in virus entry and fusion steps, not directly in virus replication (NS proteins are responsible for virus replication, not structural proteins like the E proteins)[1].

We believe our study is the first to show that Tu inhibits NS1 glycosylation and thus directly inhibits virus replication. In response to the question “it is not clear from the current data if the effect on virus replication is linked to NS1 glycosylation”, we have provided strong evidence demonstrating the requirement and significance of NS1 glycosylation for virus replication, consistent with above-mentioned reports from other groups. This evidence includes ZIKV replicon assay and plaque assay (using ZIKV infectious clone) with NS1 glycosylation site mutations, as well as ER morphology assays. Importantly, these experiments were conducted independent of Tu treatment. Additionally, Tu treatment, which blocks NS1 glycosylation, inhibited viral replication, further supporting our conclusion.

Furthermore, we did not discount the possibility that inhibitory effect of Tu on virus replication might come from the glycosylation deficiency of other NS proteins (such as NS4B) and even host proteins. Therefore, we refrained from claiming that the glycosylation deficiency of NS1 is the sole reason for Tu mediated flavivirus inhibition. Instead, we believe that NS1 glycosylation deficiency represents an important mechanism of Tu’ inhibition. We have cited the two references in the discussion section of our manuscript (Ref. 44 and 46).

4. Considering the points outlined above, the authors should either demonstrate a direct role in virus replication complex formation or change the wording throughout (including the title) to indicate that NS1 glycosylation is likely not directly involved in virus replication.

Thanks for the comment. We think we have rationally answered the points raised above with evidence demonstrating that glycosylation of NS1 is directly involved in replication compartments (RCs) formation and viral replication. Despite the fact that hypo- and non-glycosylated NS1 still have membrane binding activity, the defect in NS1 stability resulted in fewer functional NS1 protein, directly impeding RCs formation and viral replication. Additionally, others studies have also reported the impact of NS1 glycosylation on viral replication[3, 7, 8].

5. The word 'deglycosylation' is used throughout the paper for sections involving NS1 mutants. Deglycosylation would imply that sugar modifications are removed from the protein after addition. In this case, they were never added. This phrasing should be changed throughout.

Thanks for your suggestion. We have replaced ‘deglycosylation’ with ‘hypo-glycosylation’, ‘non-glycosylation’ or ‘glycosylation deficiency’ according to your suggestion.

6. In Figure 1C the authors claim that NS1 expression alters ER morphology. An ER resident marker protein should be included in these images to show ER morphology.

Thanks for your suggestion. Accordingly, we used Climp63 as an ER marker and replaced Fig. 1C, 5D and Fig. S1B in our revised manuscript (also seen as Figure 6 below). The co-localization of NS1 and Climp63 indicates that NS1 is indeed located in the ER and possesses the ability for ER remodeling.

**Figure 6. NS1 remodels ER using Climp63 as the ER marker**

**(A and B)** HeLa cells expressing Myc-tagged ZIKV NS1 **(A)** or DENV NS1 **(B)** were stained with Myc and Climp63 antibodies. NS1-positive cells were categorized into two groups based on ER morphology. Arrows indicate the aggregated ER, arrowheads indicate the normal ER. Scale bar, 20 μm. **(C)** ZIKV NS1 induced ER morphology changes upon Tu treatment, Climp63 as the ER marker. HeLa cells were transfected with Myc-tagged WT ZIKV NS1 plasmid, followed by treatment with 0.5 μg/mL Tu. Cells were fixed and stained with Myc and Climp63 antibodies. As a control, HeLa cells without transfection were treated with 0.5 μg/mL Tu (left panel). Arrows indicate cells with aggregated ER, arrowheads indicate cells with normal ER. Scale bar, 20 μm.

7. Quantification for Electron microscopy images is needed to interpret these data. Eg. What portion of cells have the indicated phenotype?

Thanks for the suggestion. Accordingly, we have added quantitative analysis for the TEM images in Fig. 1E, 5F, and 6E in the revised manuscript. The percentage of cells displaying indicated ER morphologies among 50 cells (including transfected and non-transfected cells) was quantified and listed in the table. The results showed wide-type NS1 protein induced disordered ER in more than 20% cells, whereas mutated NS1 proteins led to a reduction of cells containing disordered ER (Figure 7A and 7B). In the TEM images illustrating the effect of tunicamycin (Tu) on NS1 induced ER remodeling, our observations indicated a decrease in disordered ER from 24% to 12% after Tu treatment (Figure 7C and 7D). In the TEM images of depicting the impact of Tu on viral RCs formation, the results demonstrated a significantly inhibition of RCs formation by Tu (Figure 7E and 7F).

**Figure 7. Quantification for TEM images**

**(A)** The ultrastructure of the ER in HeLa cells overexpressing WT or mutated ZIKV NS1 was captured by TEM. Ctrl, untransfected cells. Scale bar, 1 μm (upper), 500 nm (lower). **(B)** Quantification of TEM images in (A). The percentage of cells displaying specific ER morphologies among 50 cells was determined. **(C)** TEM images showed the ER ultrastructure in HeLa cells treated with 0.25 μg/mL Tu or transfected with WT ZIKV NS1 plasmid and treated with 0.25 μg/mL Tu. Scale bar, 1 μm (upper), 500 nm (lower). **(D)** Quantification of TEM images in (C). The percentage of cells displaying specific ER morphologies upon transfection with the ZIKV NS1 plasmid among 50 cells was determined. (**E**) Tu inhibited ZIKV RCs formation. TEM images showed the ER ultrastructure in BHK-21 cells treated with 0.25 μg/mL Tu or infected with ZIKV and treated with 0.25 μg/mL Tu. Scale bar, 1 μm (upper), 500 nm (lower). **(F)** Quantification of TEM images in (E). The percentage of cells containing RCs upon infection with ZIKV among 50 cells was determined.

8. Cell viability controls should be included in the Tunicamycin experiments

Thanks for this helpful suggestion. We have performed the cell viability assay for tunicamycin (Tu) treatment in HeLa and BHK-21 cells. The cell viability of DMSO was set at 100%, and cell viability decreased in a dose-dependent manner (Figure 8). In our study, we used 0.25 μg/mL and 0.5 μg/mL of Tu, with the cell viability above 80% (Figure 8).

**Figure 8. Cell viability of HeLa cells and BHK-21 cells for tunicamycin treatment**

HeLa cells or BHK-21 cells were seeded into a 96-well clear microplate. After 24 h, the cells were treated with different concentrations of tunicamycin. Cell viability was assessed using the Cell Counting Kit-8 (Dojindo) at 12 h post treatment. The data represent mean ± SEM (n=3).

Reviewer # 3:

Majors:

1. How was quantified or normalized the amount of sample loaded on the protein gels both for Coomassie and WBs? Based on a previous total protein quantification? by volume loaded? by the amount of lyzed cells? Please, specify in each case. If this data is not indicated or was not made, the quantification made using the captured image of the gels is not feasible. This data needs to be included in the M&M section.

Thank you for this detailed suggestion. We have added the methods for quantification of protein samples in the Materials and Methods section (p.7, lines 162-181 in the manuscript).

“**Protein quantification**

For Western blotting experiments, all samples were processed consistently. Equal numbers of cells were seeded in 24-well plates and lysed with the same volume of lysis buffer. Identical volumes of samples were then loaded onto the gels. In detail, for Fig. 1A and 5A, the cell medium was replaced with 300 μL Opti-MEM, and the cells were lysed with 100 μL lysis buffer. 6 μL of cell lysate and 18 μL of medium samples were loaded onto the gel. The percentage of secreted NS1 was determined by dividing secreted NS1 by the total NS1 (the sum of intracellular and secreted NS1). For Fig. 2E and 5H, the cells were lysed with 100 μL lysis buffer and centrifuged to separate the supernatant and pellet fractions. The pellet was resuspended with 100 μL 1×SDS-PAGE loading buffer, and 10 μL of supernatant and pellet samples were loaded onto the gel. The percentage of insoluble NS1 was calculated by dividing insoluble NS1 in the pellet by the total NS1 (the sum of soluble and insoluble NS1). For Fig. 3A, the cells in 500 μL PBS were sonicated and centrifuged to pellet the membrane-associated proteins. The pellet was resuspended with 500 μL PBS, and 10 μL of supernatant and pellet samples were loaded onto the gel. For Coomassie blue staining, the total protein concentration was measured using a Nanodrop instrument (Thermo), and the NS1 protein was quantified with a BSA standard by SDS-PAGE. Therefore, for Fig. 2C, equal amount of NS1 proteins were loaded as input and the percentage of floating-up NS1 was calculated by dividing float-up NS1 by the input.”

2. In the context of the RCs formation: the authors conclude in general that the deglycosidation of NS1 affect the formation of the RCs and hence influence the viral replication. However, the experiments aimed to assay the RCs formation by TEM, were carried out only in Hela cells with recombinant NS1 proteins, while the experiments aimed to evaluate the hampering of the viral replication were made in BHK cells. To make a feasible comparison and matching of the results, I strongly suggest performing both experiments in the same cell line.

Thanks for this suggestion. Following your advice, we conducted experiments involving recombinant NS1 proteins inducing ER remodeling in BHK-21 cells. Similar ER morphology changes have been observed in both HeLa and BHK-21 cells (Figure 9).

**Figure 9. NS1 induced ER remodeling in BHK-21 cells**

BHK-21 cells were transfected with ZIKV NS1 plasmids. At 24 hpt, the cells were fixed and prepared as TEM samples. The ultrastructure of the ER in the BHK-21 cells was captured by TEM. Scale bar, 1 μm (upper), 500 nm (lower).

3. Results Section. Related to figure 1A, lanes 13-17: Authors: "Except for the double mutant, secreted NS1 proteins had larger molecular weights than their intracellular counterparts suggesting hyperglycosylation during the secretion pathway…". Comment: It is well known that the trimming process that suffer the glycan molecules throughout their transit from the ER-Golgi complex-extracellular compartment, render smaller glycans molecules. So, may you offer a different or a complementary explanation to support the differences observed in the molecular weights of the secreted NS1 proteins?

Thanks for the comment. Yes, the trimming process occurs during transit from the ER-Golgi complex-extracellular compartment. Moreover, more broad and complex protein glycosylation modifications occur in the Golgi compartment via adding further monosaccharides to growing oligosaccharide chains by glycosyltransferases, resulting in core extension, elongation and branching, and final capping of glycans. Indeed, the NS1 protein undergoes glycosylation with high mannose type sugar chains in the ER, and the N130 site is further modified into complex type by Golgi-resident enzymes in the Golgi[9, 10], resulting in more glycan molecules being added to NS1 and further increasing its molecular weight. Therefore, the molecular weights of secreted WT NS1 and NS1 N207A are larger than that of intracellular NS1 (p.11, lines 300-102 in the manuscript).

4. Results section Authors: "As anticipated, we found that tunicamycin blocked NS1 glycosylation in ZIKV- and DENV-infected cells (Figure 6F and S4D). These findings unveil the underlying mechanism by which tunicamycin inhibits flavivirus replication and validated the pivotal role of NS1 glycosylation in flavivirus RCs formation…". Comment: since Tunicamycin has a general effect blocking the glycosylation of all the viral and cellular proteins, the conclusions made from these experiments need to be scale down. It is crucial the inclusion of a control of cells treated with Tunicamycin without plasmids or infectious clones' transfections. (Fig 5, 6 and S3).

Thanks for this helpful suggestion. As mentioned in the comment, tunicamycin (Tu) can inhibit the glycosylation of other viral and host proteins, so the inhibitory effect of Tu on viral replication cannot rule out the contribution of glycosylation defects in other proteins. We have addressed this concern in the manuscript. However, the glycosylation site mutations of NS1 on replicon and infectious clone could specifically elucidate the effect of NS1 glycosylation on RCs formation and ZIKV replication.

Following your suggestion, we have added controls for Tu experiments. HeLa and BHK-21 cells were treated with either DMSO or 0.5 μg/mL Tu without plasmid transfections or virus infections (see Fig. 5D, 5F, 6E and Fig. S4D in the revised manuscript). The results indicated that Tu has no effect on ER morphology in both HeLa and BHK-21 cells.

5. Conclusions section. Authors: "… Our study unravels a novel mechanism wherein tunicamycin directly inhibits flavivirus replication by blocking NS protein glycosylation and interfering with viral RCs formation…". Based on the results of the Tunycamicin experiments without the inclusion of the proper controls, this conclusion represents an overinterpretation of the data.

Thanks for your comment. We have added controls for tunicamycin (Tu) experiments, in which the cells were treated with either DMSO or Tu without transfections or infections. Considering that Tu alone cannot affect the ER morphology but inhibit the glycosylation of NS1 protein and viral RCs formation in flavivirus infected cells, we think our conclusion is rational.

Minors:

1. It is too uncomfortable to let the comments flow because the interrupted numeration every 65 lines.

We are sorry for the typesetting problem, and we have added page numbers to the manuscript to facilitate commenting.

2. Lanes 49 and 50 (of the Liposome co-floating assay): "1μg of NS1 protein…". Specify de origin of the NS1

Thanks for this suggestion. We have modified the sentence in the manuscript: “1 μg of NS1 protein purified from HEK293T cells…” (p.8, lines 206-207 in the manuscript).

3. Lanes 4-5 (Analysis of detergent soluble and insoluble fraction). "…HeLa cells transfected with NS1 plasmids were rinsed with PBS and then lysed by RIPA buffer…" The cells were rinsed and lysed immediately after transfection? Please, specify.

We apologize for the imprecise writing and thanks for the suggestion. In fact, the cells were rinsed and lysed at 24 h post transfection. We have revised the sentence in the manuscript: “HeLa cells were transfected with NS1 plasmids. At 24 hours post-transfection (hpt), the cells were rinsed with PBS and then lysed by RIPA buffer…” (p.8, lines 215-216 in the manuscript).

4. Figure 1A. Indicate the cell line used to produce the WT and mutated NS1 proteins, in both the results and legend of the figure.

Thanks for this suggestion. We have specified the cell line for Fig. 1A in the manuscript. In the results section, we have modified the sentence to “Due to distinct glycosylation modifications, wild type (WT) and mutated NS1 from HeLa cells exhibited different molecular weights in Western blotting.” (p.11, lines 293-295 in the manuscript). In the legend section, we have revised the sentence to “Intracellular and secreted ZIKV NS1 expressed in HeLa cells were analyzed by Western blotting.” (p.24, lines 665-666 in the manuscript).

5. Fig 2:

5-1. -Legend Fig 2A: specify the cell line.

Thanks for this suggestion. We used HEK293T cells for NS1 proteins purification in Fig. 2A in the manuscript and we have specified the cell line in the legend of the Figure: “The NS1 proteins with Twin-Strep-Tags, purified from HEK293T cells, were analyzed by Coomassie blue staining.” (p.25, lines 683-684 in the manuscript).

5-2. -Fig 2A and Fig 2E: The molecular weights of the mutated NS1 proteins N130 and N207 looks at different between them in Fig 2A. However, in Fig 2E they show a very close molecular weight between them from both supernatant and pellet fractions. How can the authors explain these differences?

Thanks for this comment. The slight difference in molecular weights between the N130A and N207A mutants is attributed to variations in the glycans at the N130 and N207 sites of the NS1 protein. For Fig. 2A and Fig. 2E in the manuscript, either the difference of gel concentration or the running time of SDS-PAGE could have caused the differing distance between N130 and N207 bands. As a result, you may notice that the distance between 55 and 43 kDa markers is about 1.6 cm in Fig. 2A, whereas it is about 1.3 cm in Fig. 2E. Additionally, NS1 proteins in Fig. 2A were purified from HEK293T cells, while those in Fig. 2E came from HeLa cells. Different cell types may also contribute to slight difference. Consequently, the discrepancy in molecular weights is not a problem.

5-3. -Legend of Fig 2E. Authors: "…HeLa cells were transfected with WT or mutated ZIKV NS1…". The authors need to be more careful about the expressions used along the text to refer of cells transfected with plasmids encoding for XX protein, the cells are not transfected with proteins but plasmids.

Thanks for this suggestion. We feel sorry for this mistake and we modified the description according to the suggestion: “HeLa cells were transfected with plasmids encoding for WT or mutated ZIKV NS1 proteins, …” (p.25, lines 689-690 in the manuscript). We have checked all descriptions about transfections in the manuscript and made the necessary modifications based on your recommendation.

6. Why the authors use antibodies anti Myc instead specific NS1 antibodies to demonstrate the presence of NS1 in some experiments? (Ex. Fig 5)

Thanks for the question. The NS1 (ZIKV and DENV) expressing clones carry a Myc tag at the N-terminus. We have conducted a Western blotting assay to demonstrate that both the Myc and NS1 antibodies can specifically recognize glycosylated and non-glycosylated NS1 proteins (Figure 10). The Myc antibody is widely used and sensitive, capable of detecting the tagged NS1 proteins. Moreover, using the Myc antibody allow us to detect both ZIKV N-Myc-NS1 and DENV N-Myc-NS1 in our experiments without changing antibodies, ensuring consistency in studying shared properties of flavivirus NS1 proteins. Although we also used a DENV NS1 antibody (Biofront, Cat# BF-1191-46), which could recognize ZIKV NS1 as well, there are differences in sensitivity when detecting ZIKV and DENV NS1 using this antibody. Therefore, we used the Myc antibody in experiments involving the transfections of Myc-NS1 plasmids (ZIKV and DENV NS1), while the DENV NS1 antibody was used in experiments involving viral infection.

**Figure 10. Both Myc and NS1 antibodies can detect the NS1 protein with a Myc tag**

HeLa cells were transfected with WT ZIKV NS1 plasmid and treated with either DMSO or 0.5 μg/mL Tu at 8 hpt. Cells were harvested at 24 hpt and Western blotting was performed using the anti-NS1 and anti-Myc antibodies.

7. Please, review all the legend of the figures to be sure each specific used cell lines are included.

Thanks for this detailed suggestion. According to the suggestion, we have reviewed all the figure legends and specified the cell line used in each figure in the manuscript. HeLa cells were used in Fig. 3F, Fig. 5A, Fig. S1A, Fig. S2C and Fig. S4A. BHK-21 cells were used in Fig. 6D, Fig. 6G, Fig. S5B and Fig. S5D.

8. Fig S3B and similar: Is it not supposed that the sum of the intracellular and secreted NS1 from the DMSO control is the 100%? Please, may you clarify this?

Sorry for the confusion. In Fig. 5B and Fig. S4B in the revised manuscript (Fig. S3B in the original manuscript), the percentage of glycosylated NS1 was calculated by dividing intracellular glycosylated NS1 by the total intracellular NS1 (the sum of glycosylated and non-glycosylated NS1 in the cells). For example, in the 0.05 μg/mL Tu group, to calculated the percentage of glycosylated NS1, we measured the amount of glycosylated NS1 (top band, 2^nd^ Lane) and non-glycosylated NS1 (bottom band, 2^nd^ Lane). Then the percentage is calculated as top band / (top band + bottom band) *100%. This calculation specifically accounts for intracellular glycosylated NS1 of the sample, excluding secreted glycosylated NS1. In Fig. 5C and Fig. S4C in the revised manuscript, the percentage of secreted NS1 was determined by dividing secreted NS1 by the total NS1 (the sum of intracellular and secreted NS1). Absolute values were presented, and DMSO was not used as a 100% reference.

**References:**

1.	Wen, D., et al., *N-glycosylation of Viral E Protein Is the Determinant for Vector Midgut Invasion by Flaviviruses.* mBio, 2018. **9**(1).

2.	Ci, Y., et al., *Zika NS1-induced ER remodeling is essential for viral replication.* J Cell Biol, 2020. **219**(2).

3.	Richner, J.M., et al., *Vaccine Mediated Protection Against Zika Virus-Induced Congenital Disease.* Cell, 2017. **170**(2): p. 273-283 e12.

4.	Muylaert, I.R., et al., *Mutagenesis of the N-linked glycosylation sites of the yellow fever virus NS1 protein: effects on virus replication and mouse neurovirulence.* Virology, 1996. **222**(1): p. 159-68.

5.	Whiteman, M.C., et al., *Attenuated West Nile virus mutant NS1130-132QQA/175A/207A exhibits virus-induced ultrastructural changes and accumulation of protein in the endoplasmic reticulum.* J Virol, 2015. **89**(2): p. 1474-8.

6.	Ci, Y., et al., *Bortezomib inhibits ZIKV/DENV by interfering with viral polyprotein cleavage via the ERAD pathway.* Cell Chem Biol, 2023. **30**(5): p. 527-539 e5.

7.	Tajima, S., T. Takasaki, and I. Kurane, *Characterization of Asn130-to-Ala mutant of dengue type 1 virus NS1 protein.* Virus Genes, 2008. **36**(2): p. 323-9.

8.	Pryor, M.J., et al., *Growth restriction of dengue virus type 2 by site-specific mutagenesis of virus-encoded glycoproteins.* J Gen Virol, 1998. **79 ( Pt 11)**: p. 2631-9.

9.	Yap, S.S.L., et al., *Dengue Virus Glycosylation: What Do We Know?* Front Microbiol, 2017. **8**: p. 1415.

10.	Somnuke, P., et al., *N-linked glycosylation of dengue virus NS1 protein modulates secretion, cell-surface expression, hexamer stability, and interactions with human complement.* Virology, 2011. **413**(2): p. 253-64.
